# Structure- and Morphology-Controlled Synthesis of Hexagonal Ni_2–*x*_Zn_*x*_P Nanocrystals and Their Composition-Dependent Electrocatalytic Activity for Hydrogen Evolution Reaction

**DOI:** 10.1021/acsaem.4c00539

**Published:** 2024-07-05

**Authors:** Lisa S. Graves, Rajib Sarkar, Jordon Baker, Ka Un Lao, Indika U. Arachchige

**Affiliations:** Department of Chemistry, Virginia Commonwealth University, Richmond, Virginia 23284-2006, United States

**Keywords:** water electrolysis, hydrogen evolution reaction, transition metal phosphides, bimetallic nanostructures, electrocatalysis

## Abstract

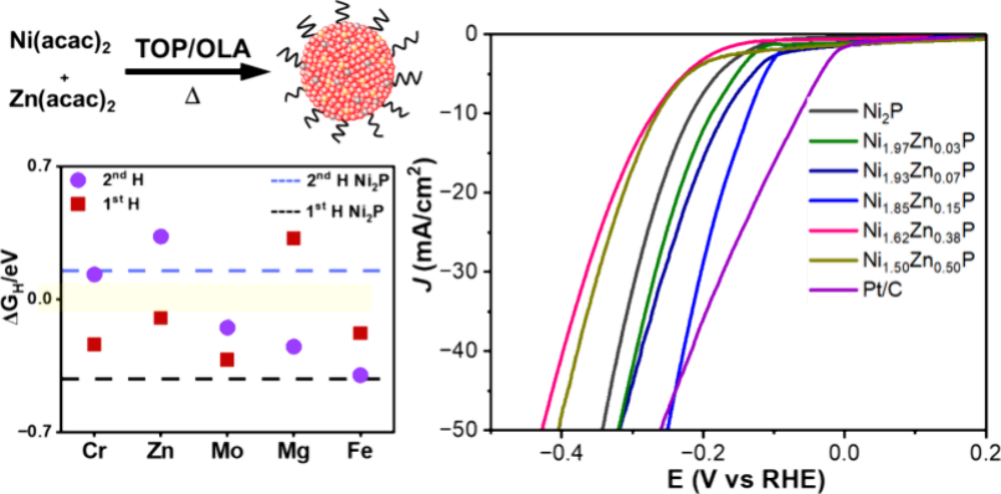

Nickel phosphides are an emerging class of earth-abundant catalysts for hydrogen generation through water electrolysis. However, the hydrogen evolution reaction (HER) activity of Ni_2_P is lower than that of benchmark Pt group catalysts. To address this limitation, an integrated theoretical and experimental study was performed to enhance the HER activity and stability of hexagonal Ni_2_P through doping with synergistic transition metals. Among the nine dopants computationally studied, zinc emerged as an ideal candidate due to its ability to modulate the hydrogen binding free energy (Δ*G*_H_) closer to a thermoneutral value. Consequently, phase pure hexagonal Ni_2–*x*_Zn_*x*_P nanocrystals (NCs) with a solid spherical morphology, variable compositions (*x* = 0–17.14%), and size in the range of 6.8 ± 1.1–9.1 ± 1.1 nm were colloidally synthesized to investigate the HER activity and stability in alkaline electrolytes. As predicted, the HER performance was observed to be composition-dependent with Zn compositions (*x*) of 0.03, 0.07, and 0.15 demonstrating superior activity with overpotentials (η_–10_) of 188.67, 170.01, and 135.35 mV, respectively at a current density of −10 mA/cm^2^, in comparison to Ni_2_P NCs (216.2 ± 4.4 mV). Conversely, Ni_2–*x*_Zn_*x*_P NCs with *x* = 0.01, 0.38, 0.44, and 0.50 compositions showed a notable decrease in HER activity, with corresponding η_–10_ of 225.3 ± 3.2, 269.9 ± 4.3, 276.4 ± 3.7 and 263.9 ± 4.9 mV, respectively. The highest HER active catalyst was determined to be Ni_1.85_Zn_0.15_P NCs, featuring a Zn concentration of 5.24%, consistent with composition-dependent Δ*G*_H_ calculations. The highest performing Ni_1.85_Zn_0.15_P NCs displayed a Heyrovsky HER mechanism, enhanced kinetics and electrochemically active surface area (ECSA), and superior corrosion tolerance with a negligible increase of η_–10_ after 10 h of continuous HER. This study provides critical insights into enhancing the performance of metal phosphides through doping-induced electronic structure variation, paving the way for the design of high-efficiency and durable nanostructures for heterogeneous catalytic studies.

## Introduction

Hydrogen generation via electrocatalysis-enabled water splitting offers an exciting opportunity to decrease our reliance on fossil fuels and address critical issues with energy sustainability. However, large-scale hydrogen production is hindered by the availability of earth-abundant catalysts with comparable or higher activity to expensive noble metal catalysts. Nickel phosphides have emerged as a burgeoning noble metal free alternative for the hydrogen evolution reaction (HER) because of high activity and stability compared to other nonnoble metal catalysts.^1−3^ However, they do not exhibit low overpotentials (η) for HER and oxygen (OER) evolution and high corrosion tolerance in acid and alkali compared to noble metal catalysts. These deficiencies stem from the limited tunability of surface affinity and adsorption strength of metal–hydrogen intermediates for monometallic phosphides;^4^ however, doping with synergistic transition metals can be employed to substantially improve the stability and electrocatalytic performance.^5−10^

The crystal structure of Ni_2_P comprises two alternating stoichiometric planes, Ni_3_P and Ni_3_P_2_, along the [0001] direction.^11^ The Ni-rich Ni_3_P_2_ surface, exposed by the Ni_2_P (001) facet, is the most active surface for reversible binding of hydrogen, providing highly active Ni sites (hydride-acceptor) and less active P sites (proton-acceptor) for the water splitting reaction.^12^ However, the catalytic activity of the Ni_2_P (001) surface is not optimal for water splitting, because the Ni sites bond strongly to hydrogen, impeding the facile removal of H_2_.^12^ Therefore, an increase in surface metal sites along with systematic modulation of the surface affinity is needed to further improve the HER activity, which can be achieved via admixing of synergistic metal atoms. To understand how different dopants and doping-induced structures impact the HER activity for designing efficient electrocatalysts, theoretical calculations are essential. These provide insights into structural modulation at the atomic level and have been proven useful for guiding experiments.^12−15^ However, to the best of our knowledge, only a handful of computational studies focused primarily on late transition metals have investigated the effect of dopants on the HER activity and stability of the Ni_2_P (001) surface.^16−20^

Recent literature indicates that nickel phosphides doped with V,^14,21,22^ W,^23^ Al,^24^ Mo,^25^ Fe,^26^ Co,^26^ and Mn^27^ show improved HER performance in comparison to monometallic counterparts. For instance, V-doped Ni_2_P nanosheets (NS) showed a notable increase in HER activity with an overpotential (η) of 85 mV compared to Ni_2_P NS (143 mV) at a current density (*j*) of −10 mA/cm^2^ (η_–10_).^21^ Similarly, Mn-doped Ni_2_P NS showed enhanced HER performance (η_–20_ = 185 mV) in comparison to Ni_2_P NS (η_–20_ = 103 mV).^27^ A composition-dependent study exploring the HER activity of Ni_2–*x*_Mo_*x*_P nanocrystals (NCs) reported a prominent increase in HER performance for homogeneously alloyed Ni_1.87_Mo_0.13_P NCs (η_–10=_ 101 mV) in comparison to Ni_2_P NCs (244 mV).^13^ Interestingly, the larger heterogeneous Ni_2–*x*_Mo_*x*_P NCs displaying the same dopant concentration (i.e., Ni_1.87_Mo_0.13_P) showed a higher HER activity (η_–10_ = 96 mV) in comparison to heterogeneous Ni_2_P NCs (η_–10_ = 156 mV).^28^ In both cases, the Ni_1.87_Mo_0.13_P composition displayed the lowest Tafel slopes, which were comparable to benchmark Pt/C catalyst, suggesting a pronounced increase in HER kinetics upon heteroatom doping. These reports illustrate the importance of optimal dopants and their critical concentration for modulating the surface affinity of nickel phosphides in the development of high-efficiency and earth-abundant HER electrocatalysts. Although the influence early transition metal dopants have been extensively studied, a few experimental studies on late transition metal-doped Ni_2_P for HER have been reported.^20,29^ Moreover, a systematic study on the influence of late transition metal dopants *(*i.e., Zn and Cu) on HER activity and stability of nickel phosphides, to our knowledge, has not been reported. This work aims to increase the arsenal of transition metal-doped Ni_2_P catalysts by investigating the composition-dependent effects on the HER activity and stability of discrete Zn-doped Ni_2_P NCs.

Herein, a comprehensive computational analysis of nine synergistic dopants (Cr, Zn, Mo, Mg, Fe, V, Cu, Ti, and W) was conducted to evaluate their influence on the hydrogen adsorption free energy (Δ*G*_H_) on the hexagonal Ni_2_P (001) surface. Density functional theory (DFT) analysis indicates Zn as an optimal dopant that promotes H adsorption and desorption near the thermoneutral Δ*G*_H_ on more energetically favorable binding sites. Accordingly, a series of phase pure Ni_2–*x*_Zn_*x*_P NCs with hexagonal crystal structure, solid spherical morphology, and variable compositions were synthesized to investigate the compositional effects on HER activity in alkaline electrolytes. Binary Ni_2_P NCs displayed η_–10_ of 216.2 ± 4.4 mV, consistent with literature reports.^28^ In contrast, Ni_2–*x*_Zn_*x*_P NCs showed significantly improved HER performance and a progressive decrease of η_–10_ from 188.7 ± 3.7, 170.0 ± 4.2, and 135.4 ± 3.4 mV for *x* = 0.03, 0.07, and 0.15 compositions, respectively. At concentrations above 5.24% Zn (*x* = 0.15), bimetallic NCs showed a notable decrease in HER activity compared to monometallic Ni_2_P NCs. The catalyst with the highest HER activity was determined to be Ni_1.85_Zn_0.15_P NCs, consistent with composition-dependent Δ*G*_H_ calculations. The highest performing Ni_1.85_Zn_0.15_P NCs outperformed the HER activity of benchmark Pt/C (10% wt.) catalyst at *j* >−45 mA/cm^2^. Moreover, the stability of Ni_1.85_Zn_0.15_P NCs was superior to that of Ni_2_P NCs and Pt/C catalyst, where Ni_1.85_Zn_0.15_P NCs showed a 4.87% increase in η_–10_ after 10 h of HER in comparison to 29.92 and 58.79% increases noted for Ni_2_P and Pt/C electrocatalysts, respectively.

## Experimental Section

### Materials

Nickel(II) 2,4-pentanedionate (Ni(acac)_2_, 95%) and bis(2,4-pentanedionato), zinc(II) (Zn(acac)_2_, 96.0%), oleylamine (OLA, 70%), toluene, and ethanol were purchased from Fisher. Tri-n-octylphosphine (TOP, 97%) was purchased from Strem Chemicals. Carbon-coated 200 mesh copper grids were purchased from SPI Supplies. Titanium foil (thickness 0.25 mm; 99.7%) and platinum on graphitized carbon (Pt/C, 10% wt.) were purchased from Sigma-Aldrich. Graphite rods (6.15 mm × 102 mm, 99.9995%) were purchased from Alfa Aesar. Hg/HgO reference electrode filled with 1 M NaOH was purchased from CH Instruments. Pelco colloidal silver paint was purchased from Ted Pella. Henkel Loctite EA-9462 epoxy adhesive was purchased from Ellsworth Adhesives. Chemical-resistant PTFE-insulated silver plated-copper wire was purchased from McMaster-Carr. OLA was dried under vacuum for 3 h prior to use. Toluene was dried over Na metal, and ethanol was dried over molecular sieves and CaO and then distilled under N_2_ prior to use.

### Synthesis of Ni_2_P NCs

The synthesis of Ni_2_P NCs was adopted from a literature procedure.^30^ Briefly, 1.0 mmol of Ni (acac)_2_, 10.0 mL of OLA, and 5.0 mL of TOP were combined in a 50 mL round-bottom flask under a nitrogen atmosphere and attached to a Schlenk line. This solution was degassed under vacuum at 120 °C for 20 min, purged with nitrogen, and the temperature was raised to 330 °C where it was kept for 2.5 h. Then, the reaction was cooled to room temperature and NCs were isolated with a mixture of toluene and ethanol and centrifuged for 10 min. This purification procedure was repeated a minimum of 3 times. The final NC product was dried under vacuum.

### Synthesis of Ni_2–*x*_Zn_*x*_P NCs

The synthesis of Ni_2–*x*_Zn_*x*_P NCs was modified from the synthesis of Ni_2_P NCs. Ni(acac)_2_ and Zn(acac)_2_ precursors were combined with the desired nominal molar ratio (Table 1) and added to a round-bottom flask containing 10.0 mL of OLA and 5.0 mL of TOP. The flask was attached to a Schlenk line, and the mixture was degassed under vacuum at 120 °C for 20 min. Then, the flask was purged with nitrogen and the temperature was increased to 330 °C. The solution was kept under reflux at 330 °C for 2.5 h, and the purification procedure remained unchanged.

**Table 1:** Nominal and Experimental Compositions as well as Crystallite and Average Particle Sizes of Hexagonal Ni_2–*x*_Zn_*x*_P NCs Produced via Colloidal Synthesis

	elemental composition (ICP-OES)^a^					
sample name	Ni	Zn	P	nominal Zn composition	crystallite size, nm (PXRD)^b^	particle size, nm (TEM)^c^
Ni_1.99_Zn_0.01_P	75.02	0.41	24.56	5%	8.0	7.4 ± 1.2
Ni_1.97_Zn_0.03_P	73.65	1.26	25.10	5%	8.8	9.1 ± 1.1
Ni_1.93_Zn_0.07_P	71.37	2.60	26.03	10%	8.0	9.1 ± 1.1
Ni_1.90_Zn_0.10_P	69.28	3.69	27.03	10%	9.1	8.9 ± 1.1
Ni_1.85_Zn_0.15_P	63.84	5.24	30.93	10%	8.2	8.9 ± 1.4
Ni_1.77_Zn_0.23_P	62.73	8.17	29.10	15%	8.1	8.5 ± 1.2
Ni_1.62_Zn_0.38_P	57.32	13.23	29.44	25%	7.7	6.8 ± 1.1
Ni_1.56_Zn_0.44_P	53.57	15.32	31.11	25%	7.8	7.3 ± 1.0
Ni_1.50_Zn_0.50_P	51.65	17.14	31.11	30%	8.4	7.1 ± 1.2

a Elemental compositions of Ni, Zn, and P were obtained by ICP-OES. Each composition was determined by averaging three individual measurements per sample.

b Crystallite size was calculated by applying the Scherer equation to the (111) reflection of the PXRD pattern.

c Average particle size was calculated from 125 to 200 individual NCs from several LR- and HR-TEM images. Corresponding size histograms are shown in the Supporting Information, Figure S2.

### Physical Characterization

Powder X-ray diffraction (PXRD) patterns were recorded using an Empyrean multipurpose X-ray diffractometer equipped with a Cu Kα (λ = 1.5418 Å) radiation source, under 45 kV and 40 mA operating conditions. The Scherrer formula was used to manually calculate the crystallite size based on the hexagonal Ni_2_P (111) reflection. Low-resolution transmission electron microscopy (TEM) images were acquired using a Jeol JEM-1400Plus transmission electron microscope equipped with a Gatan UltraScan 4000SP 4K x 4K CCD camera, operating at 120 kV. High-resolution TEM, scanning (STEM) images, and elemental maps were attained using a JEM-F200 cold FEG electron microscope operating at a voltage of 200 kV and equipped with an energy-dispersive X-ray analyzer. Sample preparation for TEM/high-resolution (HR)TEM involved drop-casting a 10 μL aliquot of a dilute Ni_2–*x*_Zn_*x*_ P NC-toluene solution onto carbon-coated copper grids and toluene was evaporated in ambient air prior to imaging. Elemental compositions were determined through inductively coupled plasma-optical emission spectroscopy (ICP-OES), using an Agilent Technologies 5110 ICP-OES equipped with a SPS4 autosampler. Samples were digested in acid (3:1 v:v HCl/HNO_3_) and placed in a heated water bath (∼60 °C) for 24 h and then further diluted with Milli-Q-filtered water prior to analysis. X-Ray photoelectron spectra (XPS) were recorded using a PHI VersaProbe III Scanning XPS Microprobe. NCs were annealed in 5% H_2_:Ar at 450 °C for 2 h prior to XPS analysis. Regional scans were completed using a pass energy of 26.00 eV with 20 ms per step, and the number of sweeps was dependent upon the signal intensity of each element. FT-IR spectra were recorded using a Thermo Scientific Nicolet iS50 FT-IR.

### Theoretical Calculations

To investigate the most effective metal-doped nickel phosphides for HER, a comprehensive study on the Ni_2_P (001) surface doped with a series of synergistic metal (Cr, Zn, Mo, Mg, Fe, V, Cu, Ti, and W) atoms was conducted using density functional theory (DFT) within the Vienna *Ab initio* Simulation Package (VASP)^31^ and the Projector-Augmented-Wave (PAW) method. The electrocatalytic performance was assessed by calculating the Δ*G*_H_. Utilizing the Bell–Evans–Polanyi principle, we focused on Δ*G* binding interactions, allowing us to bypass the computationally demanding calculations of reaction barriers.^15^ The Δ*G*_H_ at the hydrogen adsorption site was considered a critical descriptor of each catalyst’s intrinsic activity.^12^ An ideal catalyst is designed with a Δ*G*_H_ value close to 0 eV, aligning with the Sabatier principle, ensuring that hydrogen binds neither too weakly nor too strongly to the surface.^32^ Therefore, smaller |Δ*G*_H_| values indicate a higher HER activity.^33^ Because of the influence of Δ*G*_H_ values from the computational level,^16−18^ |Δ*G*_H_| <0.1 eV was adopted as the optimal criterion^32^ to identify the most active heteroatom-doped Ni_2_P catalyst.

The calculation of Δ*G*_H_ was conducted using the equation:^34,35^

Δ*G*_H_ = Δ*E* + Δ*E*_ZPE_ – *T*Δ*S*.

Here, Δ*E*, Δ*E*_ZPE_, *T*, and Δ*S* indicate the electronic adsorption energy obtained through DFT, zero-point energy, temperature, and entropy contributions, respectively. A commonly utilized factor of 0.24 eV^22,36^ was applied to encompass all contributions in entropic and zero-point energy (Δ*E*_ZPE_ – *T*Δ*S*). Consequently, Δ*G*_H_ was simplified to Δ*E* + 0.24 eV. The Perdew–Burke–Ernzerhof (PBE) functional^37^ was employed to obtain structures, while PBE with the Grimme D3(BJ) dispersion correction^38,39^ was employed for Δ*E* calculations. A periodically repeated slab with six layers and a 15 Å vacuum (cell parameters *a* = *b* = 5.891 Å and *c* = 25.120 Å) was employed, with atoms at the bottom layer fixed to their bulk positions, to model Δ*G*_H_ on the Ni_2_P (001) surface with Ni_3_P_2_ termination. The emphasis on the Ni_3_P_2_ termination was due to it being the most active Ni_2_P surface termination.^40,41^ A plane wave cutoff energy of 520 eV and a Γ-centered 5 × 5 × 1 grid of k-points were utilized. In our HER calculations, one Ni atom was substituted with one of the nine doped metals to identify the most stable doping site, resulting in a 5.56% dopant concentration. We also investigated the dependence of Δ*G*_H_ on Zn concentration. For the 11.11% Zn concentration, we maintained the same cell parameters used for the 5.56% Zn concentration but replaced two Ni atoms with Zn atoms to find the most stable structure. To generate a 2.78% Zn computation, a larger cell (*a* = 11.782 Å, *b* = 5.891 Å, and *c* = 25.120 Å) was created by substituting one Ni with a Zn atom, and the most stable structure was then determined. To validate a direct comparison of Δ*G*_H_ bindings in two cells with different Zn concentrations, the first and second Δ*G*_H_ bindings on the pristine Ni_2_P (001) surface with Ni_3_P_2_ termination were also calculated. In the smaller cell, the first and second Δ*G*_H_ bindings were −0.42 and 0.15 eV, respectively. Correspondingly, for the larger cell, bindings were −0.47 and 0.15 eV, respectively. The marginal energy difference observed ensures the validity of comparing the binding energies of two cells with different Zn compositions.

### Fabrication of Working Electrodes

Working electrodes were fabricated on Ti foils using Ni_2–*x*_Zn_*x*_P NCs and commercial Pt/C catalysts. The foils were cut into rectangles (0.5 cm × 0.4 cm) and then subjected to a multistep cleaning process that includes sonication in a mixture of acetone and ethanol (1:1, v:v) for 10 min, soaking in a mixture of 1 M HCl and 30% H_2_O_2_ (1:1, v:v) for 20 min, and rinsing via sonication in Milli-Q water (18 Ω). To fabricate catalyst ink, 4 mg of Ni_2–*x*_Zn_*x*_P or commercial Pt/C catalyst was mixed with 380 μL of isopropanol and 20 μL of nafion and sonicated in an ice water bath for 10 min. The catalyst ink (40 μL) was drop cast onto the Ti foil in 10 μL of aliquots. The catalyst-coated Ti foils were then annealed at 450 °C for 2 h under 5% H_2_:Ar to remove residual surface ligands. Then, foils were attached to a Ag-plated Cu wire using Ag paint, which ensures ohmic contact between the Cu wire and Ti foil. A two-part epoxy was used to cover the Cu wire and Ag paint, leaving only the catalyst area (0.20 cm^2^) exposed for HER studies. The epoxy was left dry in ambient air for 24 h prior to electrocatalytic experiments.

### Electrochemical Measurements

A CHI 760E electrochemical workstation was used to study the HER activity of Ni_2–*x*_Zn_*x*_P NCs and Pt/C in nitrogen-saturated 1 M KOH at room temperature. A conventional three-electrode electrochemical cell was employed for all experiments where a graphite rod, Hg/HgO (1 M NaOH) electrode, and Ni_2–*x*_Zn_*x*_P NCs or Pt/C-coated Ti foil were used as counter, reference, and working electrodes, respectively. Overpotentials were reported with respect to the scale of reversible hydrogen electrode (RHE) potential by using the conversion formula: *E*_RHE_ = *E*_Exp_ + *E*_Hg/HgO_^0^ + 0.05916pH. The current density (*j*, mA/cm^2^) was calculated using the geometrical surface area of the electrode (0.2 cm^2^). Working electrodes were electrochemically cleaned and activated by performing cyclic voltammetry (CV) and sweeping the potential from 0.2 to 0.1 V at different scan rates. Polarization curves were recorded using linear sweep voltammetry (LSV) by scanning the potential from 0.2004 to −0.4996 V (vs RHE) at a scan rate of 5 mV/s. The electrochemically active surface area (ECSA) was calculated using the electrochemical double layer capacitance (*C*_DL_), by following established literature methods.^6,42−44^ CVs were recorded within the non-Faradaic region at various scan rates (50, 100, 200, and 400 mV/s), which were utilized to determine the difference in double layer capacitive current. This difference in capacitive current [Δ*j* = (*j*_a_ – *j*_c_)/2] was plotted against the scan rate, and corresponding slopes were generated through linear fit analysis to determine the *C*_DL_. Finally, ECSA was calculated by dividing the *C*_DL_ with specific capacitance (*C*_s_ = 0.040 mF/cm^2^).^42,43,45^ Chronopotentiometry analysis was performed at a current density of −10 mA/cm^2^ for 10 h in N_2_-saturated 1 M KOH to investigate the stability of all catalysts.

## Results and Discussion

### Theoretical Calculations

**Figure 1:**
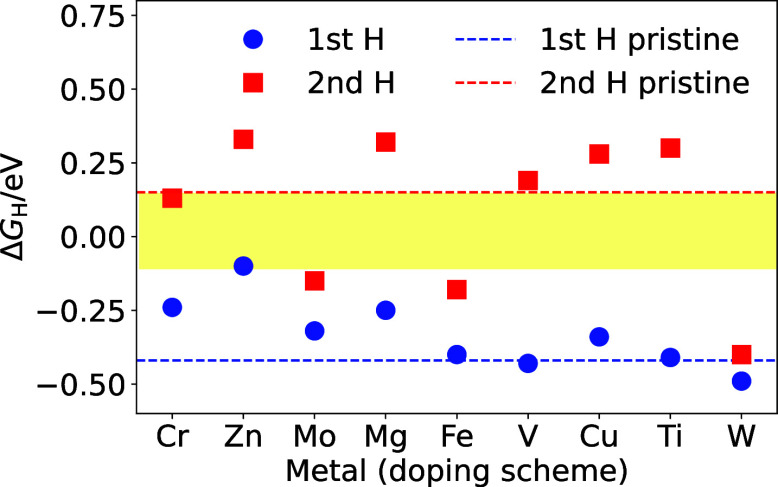
Hydrogen adsorption free energies (Δ*G*_H_) for the Cr, Zn, Mo, Mg, Fe, V, Cu, Ti, and W-doped Ni_3_P_2_-terminated Ni_2_P (001) surface calculated at the level of PBE+D3(BJ) with a plane wave cutoff energy of 520 eV and a Γ-centered 5 × 5 × 1 grid of k-points. The circles and squares indicate the lowest Δ*G*_H_ for the first and second H adsorption, respectively. The blue and red dashed lines are the lowest Δ*G*_H_ values for the first and second H adsorptions on pristine Ni_2_P, respectively. The yellow band highlights the ±0.1 eV region around the optimal Δ*G*_H_ = 0 value.

The lowest first and second Δ*G*_H_ values on Cr, Zn, Mo, Mg, Fe, V, Cu, Ti, and W-doped Ni_2_P (001) surfaces with the Ni_3_P_2_ termination are depicted in Figure 1, along with the corresponding configurations in the Supporting Information, Figure S1. The first Δ*G*_H_ on the pristine Ni_2_P surface at the Ni_3_-hollow site is −0.42 eV, which aligns with the first Δ*G*_H_ obtained in previous studies.^16,19,41^ However, such a large hydrogen binding energy indicates that pristine Ni_2_P is not catalytically active for HER at low hydrogen coverage due to the difficulty in H_2_ desorption. The second most stable hydrogen adsorption site is at the Ni–Ni bridge with a second Δ*G*_H_ of 0.15 eV, consistent with a prior report.^16^ The binding of the second hydrogen also facilitates the first hydrogen binding at the Ni–Ni bridge site. This observation indicates that the binding of both hydrogens at the Ni–Ni bridge site is more energetically favorable than one hydrogen adsorption at the Ni_3_ hollow site and another at the Ni–Ni bridge site. The Ni–Ni bridge site is more catalytically active than the Ni_3_-hollow site, and this behavior can be attributed to the enhanced HER activity of Ni_2_P reported in the literature.^13,46^ The HER activity of Ni_2_P can be enhanced by reducing Δ*G*_H_ on the Ni_3_-hollow site and increasing Δ*G*_H_ on the Ni–Ni bridge site. Therefore, the metal doping strategy, adopted in this study, could prove useful for improving the HER activity of hexagonal Ni_2_P.^2^

As shown in Figure 1, doping can substantially modify the HER activity of the Ni_3_P_2_-terminated Ni_2_P (001) surface. The most promising dopants for HER are Cr, Zn, and Mo based on the overall values for the first and second Δ*G*_H_. Specifically, Cr doping modifies the first Δ*G*_H_ from −0.42 (pristine Ni_2_P) to −0.24 eV and the second Δ*G*_H_ from 0.15 (pristine Ni_2_P) to 0.13 eV. For Cr doping, both the first and second hydrogens occupy Cr–Ni bridge sites. Doping with Zn significantly decreases the first Δ*G*_H_ to −0.10 eV, but it also results in the second Δ*G*_H_ becoming more repulsive at 0.33 eV. For Zn doping, the first hydrogen occupies the Ni–Ni bridge site, and the second hydrogen is located on the Ni–P bridge site. Similarly, Mo doping changes the first and second Δ*G*_H_ to −0.32 and −0.15 eV, respectively. For Mo doping, the first hydrogen still occupies the hollow site, but the second hydrogen locates at the Mo atop site. The other dopants (Mg, Fe, V, Cu, Ti, and W) investigated in this study did not substantially improve the Δ*G*_H_ in comparison to pristine Ni_2_P.

### Synthesis and Characterization of Ni_2_P and Ni_2–*x*_Zn_*x*_P NCs

Computational analysis of the nine dopants suggests that Zn would be a promising dopant for hexagonal Ni_2_P. Consequently, Ni_2–*x*_Zn_*x*_P NCs were synthesized to experimentally investigate the composition-dependent HER activity in 1 M KOH. The colloidal synthesis of phase pure Ni_2_P NCs involves the thermal decomposition of Ni(acac)_2_ in an OLA and TOP system, where OLA acts as a surfactant and TOP acts as both a surfactant and phosphorus precursor. By changing reaction parameters such as the starting P:Ni molar ratio, temperature, and time, various nickel phosphide phases can be targeted, where higher P:Ni ratios, higher temperatures, and longer growth times favor more phosphorus-rich nickel phosphides.^30,47−49^ Furthermore, the thermal decomposition using P/Ni molar ratios >2.24 results in Ni_*x*_P_*y*_ nucleating species, which form amorphous particles at 240 °C and then crystallize to form smaller solid particles above 300 °C.^14,32^ We found that phase pure solid Ni_2_P NCs can be reproducibly synthesized under reaction conditions of 330 °C for 2.5 h with a P:Ni ratio of 11.2. The incorporation of Zn(acac)_2_ did not impact the reaction as Ni_2–*x*_Zn_*x*_P NCs retained the hexagonal Ni_2_P structure with no evidence of elemental Zn or Zn_3_P_2_ impurity phases.

Zn-doped Ni_2_P compositions were investigated using ICP-OES, and the concentration of Zn varied from *x* = 0.01–0.50, corresponding to 0.41–17.14% experimental Zn concentrations (Table 1). The experimental atomic percent of Zn was repeatedly found to be lower than the nominal moles of Zn(acac)_2_ used in the synthesis. For instance, Ni_2–*x*_Zn_*x*_P NCs produced with a nominal 10% Zn(acac)_2_ typically yields an experimental Zn concentration ranging from 2.60 to 5.24%, whereas NCs synthesized with a nominal 15% Zn(acac)_2_ show experimental Zn concentration from 5.50 to 8.61%. In contrast, alloyed NCs with significantly higher Zn (13.23–15.32%) compositions can be targeted with a nominal Zn(acac)_2_ concentration of 25%.

**Figure 2:**
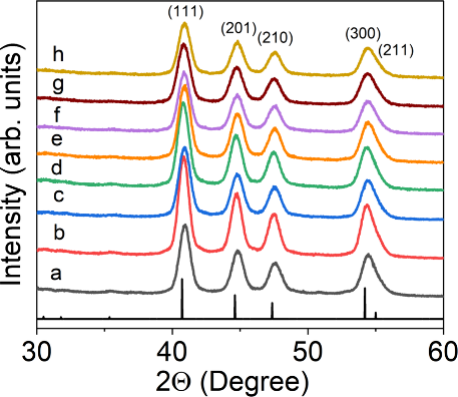
Power X-ray diffraction patterns of Ni_2_P and Ni_2–*x*_Zn_*x*_P NCs with variable Zn compositions: (a) *x* = 0, (b) *x* = 0.01, (c) *x* = 0.10, (d) *x* = 0.15, (e) *x* = 0.23, (f) *x* = 0.38, (g) *x* = 0.44, and (h) *x* = 0.50. All samples are consistent with the hexagonal Ni_2_P reference pattern (JCPDS file No. 01-074-1385) represented by vertical black lines.

Structure, crystallinity, and phase purity of Ni_2–*x*_Zn_*x*_P NCs were confirmed through PXRD, and diffraction patterns that show variable Zn compositions (0–17.14%) are shown in Figure 2. All samples displayed a hexagonal crystal structure consistent with the binary Ni_2_P reference pattern (JCPDS file No. 01-074-1385) with no evidence of impurity phases of nickel phosphides. Although no apparent shifts in the diffraction patterns were observed, slight broadening of Bragg reflections was noted and confirmed with increased full width at half maxima (fwhm) of Zn-doped samples. This observation is consistent with an earlier report, where no shifts in Bragg reflections were noted for Ni_5–*x*_Zn_*x*_P_4_ NCs because of the similar size of Ni and Zn.^50^ This combined with broader diffraction peaks obtained for Ni_2–*x*_Zn_*x*_P NCs make it more challenging to distinguish minor peak shifts. Nevertheless, the effect of Zn doping was evident in the crystallite size estimated by applying the Scherrer formula to the (111) reflection. Overall, it appears that admixing of Zn tends to decrease the crystallite size, as the size decreased from 9.0 nm for binary Ni_2_P to 8.2 and 7.8 nm for *x* = 0.15 and *x* = 0.44 compositions, respectively. However, at *x* = 0.50, crystallite size displayed a deviation from this trend and increased to 8.4 nm. The average particle size calculated from TEM images also reflects an oscillating effect with Zn concentration, where an increase was observed at lower Zn (*x* <0.15) compositions and then a decrease at higher Zn (*x* >0.15) compositions.

**Figure 3:**
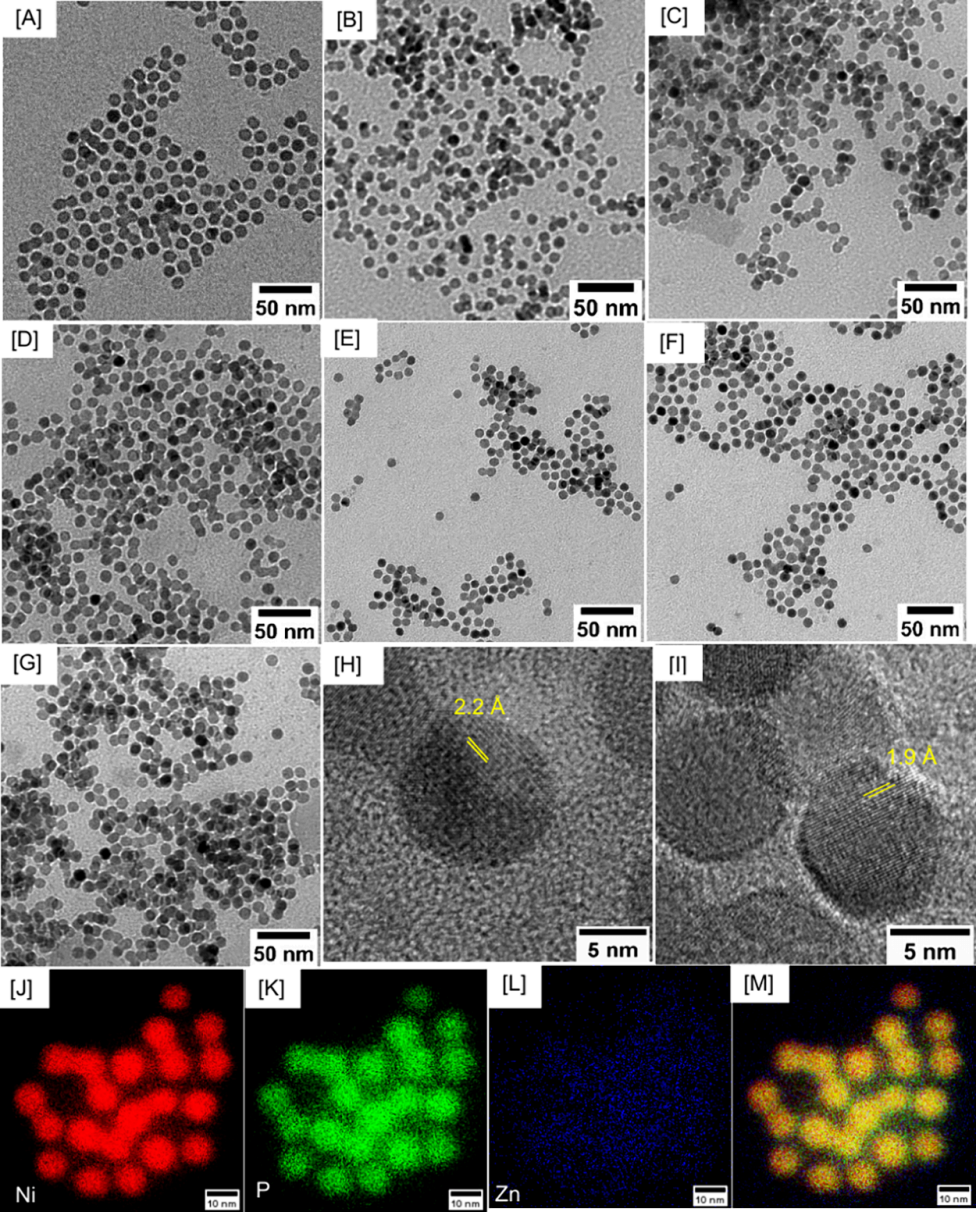
Low-resolution TEM images of hexagonal Ni_2–*x*_Zn_*x*_P NCs with variable Zn compositions: (A) *x* = 0 (8.7 ± 1.5 nm), (B) *x* = 0.01 (7.4 ± 1.2 nm), (C) *x* = 0.15 (8.9 ± 1.4 nm), (D) *x* = 0.23 (8.5 ± 1.2 nm), (E) *x* = 0.38 (6.8 ± 1.1 nm), (F) *x* = 0.44 (7.3 ± 1.0 nm), and (G) *x* = 0.50 (7.1 ± 1.2 nm). High-resolution TEM images of (H) *x* = 0 and (I) *x* = 0.50 compositions with corresponding lattice spacing measurements for the (111) reflection. STEM-EDS maps of Ni_1.77_Zn_0.23_P NCs (J) Ni, (K) P, and (L) Zn and the (M) corresponding overlay image.

TEM images of Ni_2–*x*_Zn_*x*_P NCs with variable compositions are shown in Figure 3 and in the Supporting Information, Figures S2–S4. Binary Ni_2_P NCs possessed a solid spherical morphology, which was maintained in ternary NCs with Zn content up to 17.14%. While preserving the narrow size dispersity of Ni_2_P NCs, incorporation of Zn displayed an interesting effect on the size of ternary NCs. The average size of binary Ni_2_P NCs was 8.7 ± 1.5 nm. Upon doping with *x* = 0.01, the average size decreased to 7.4 ± 1.2 nm. However, as *x* increased to 0.03, a slight increase in size to 9.1 ± 1.1 nm was observed. This trend of slightly larger particles continued with Zn compositions up to 0.15 where the average size was measured to be 8.9 ± 1.4 nm. However, beyond *x* = 0.15, the average size decreased to 8.5, 6.8, and 7.1 nm at *x* = 0.23, 0.38, and 0.50 compositions, respectively. Lattice spacing for binary Ni_2_P NCs was found to be 2.2 Å, corresponding to the (111) reflection, consistent with the literature,^47^ whereas a slightly contracted (111) lattice spacing of 1.9 Å was obtained for the highest Zn composition (*x* = 0.50). Bright field TEM images and STEM-EDS elemental maps of Ni_1.77_Zn_0.23_P NCs are shown in Figure 3J-M and in the Supporting Information, Figure S5. Elemental maps confirm the presence of Ni, Zn, and P in all samples and homogeneous admixing of all elements with no evidence of segregation. This has been confirmed with Ni_2–*x*_Zn_*x*_P NCs with variable compositions up to *x* = 0.50 (Supporting Information, Figures S6 and S7), which support no evidence of segregation at the highest Zn concentration.

**Figure 4:**
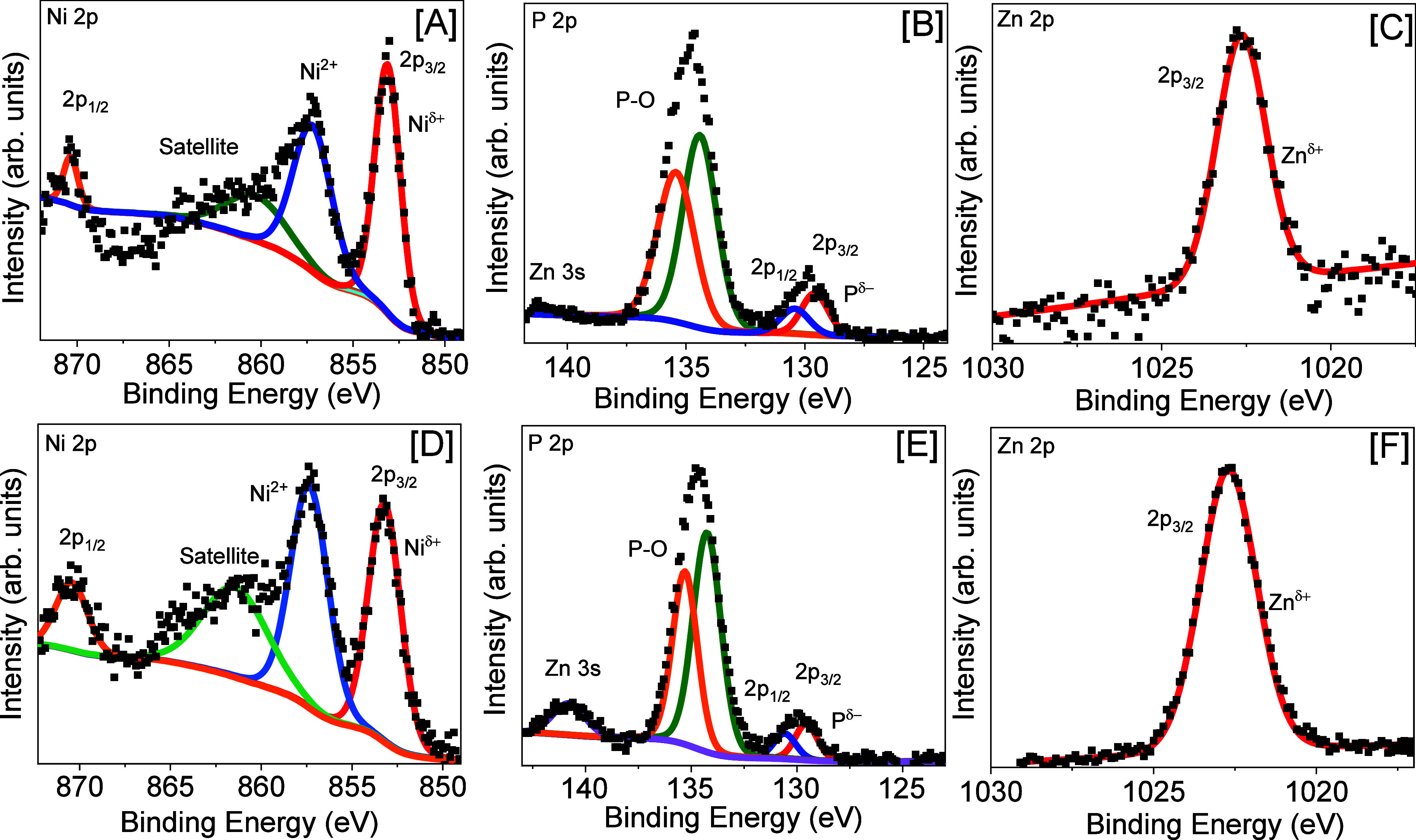
XPS spectra of Ni_1.85_Zn_0.15_P NCs showing (A) Ni 2p, (B) P 2p, and (C) Zn 2p regions along with Ni_1.56_Zn_0.44_P NCs displaying (D) Ni 2p, (E) P 2p, and (F) Zn 2 p regions. Square symbols represent experimental data, and colored lines are fitted deconvolutions. Samples were annealed for 2 h at 450 °C under a 5% H_2_:Ar atmosphere.

Surface characteristics were investigated using XPS, and spectra obtained for Ni_2_P, Ni_1.85_Zn_0.15_P, and Ni_1.56_Zn_0.44_P are shown in Figure 4 and in the Supporting Information, Figure S8. In the Ni 2p region for Ni_2_P, a partial positive charge was observed for nickel with the 2p_3/2_ peak at 852.96 eV and the 2p_1/2_ peak at 870.28 eV. Peaks at 856.43 and 856.30 eV represent the presence of NiO and Ni satellites, respectively.^28^ The P 2p region for Ni_2_P exhibits two doublets at 129.52 and 133.37 eV, which correspond to a metal phosphide^51^ and oxidized phosphorus, respectively.^28^ Ni_1.85_Zn_0.15_P NCs displayed similar characteristics with a partial positive charge on Ni represented by the Ni 2p_3/2_ peak at 853.21 eV and Ni 2p_1/2_ peak at 870.43 eV. NiO and Ni satellite moieties were also present. The metal phosphide and oxidize P doublets in the P 2p region of Ni_1.85_Zn_0.15_P NCs were demonstrated with peaks at 129.86 and 134.70 eV, respectively. Within the Zn 2p region of the Zn-doped sample, the peak at 1022.66 eV indicates a slight partial positive charge on Zn surface species.^52^ The higher Zn-doped composition, Ni_1.56_Zn_0.44_P, exhibited similar surface characteristics. Interestingly, as the Zn composition increased from *x* = 0, 0.15, to 0.44, the partial positive charge on Ni increases as the Ni 2p_3/2_ peak shifted from 852.96 to 853.21 then to 853.28 eV, respectively. P also underwent a similar change as the partial negative charge decreased by 0.44 eV, and the P^δ−^ peak shifts from 129.52 to 129.86 then to 129.96 eV, for *x* = 0, 0.18, and 0.40 compositions, respectively. Although rather dramatic shifts were observed in Ni and P surface moieties as a function of Zn concentration, the partial positive charge on Zn showed a slight shift to higher binding energies (1022.66–1022.70 eV) as the *x* increased from 0.15 to 0.44. Since Zn is less electronegative than Ni, a shift of Ni^δ+^ to lower binding energies is expected. Instead, the peaks for both Ni^δ+^ and P^δ−^ shift to higher binding energies. Furthermore, the oxidized P and Ni moieties become more pronounced as the Zn content increases from *x* = 0 to 0.44, suggesting that the presence of Zn causes Ni_2_P to be more susceptible to surface oxidation, leading to the higher binding energies observed for Ni^δ+^ and P^δ−^ peaks.

### Electrocatalytic Activity of Ni_2–*x*_Zn_*x*_P NCs for HER

The HER activity of Ni_2–*x*_Zn_*x*_P NCs was investigated using linear sweep voltammetry (LSV) in a three-electrode electrochemical cell in N_2_-saturated 1 M KOH solution. The catalytic activities of bare Ti foil and commercial Pt/C (wt. 10%) standard were also investigated for comparison. A range of solvents such as isopropanol, ethanol, isopropanol + ethanol (1:1), isopropanol + water (1:1), and ethanol + water (1:1) were used to fabricate catalyst inks both in the presence and absence of nafion and isopropanol produced consistent HER data for all samples examined. In the presence of nafion, catalysts showed the highest HER activity as it prevents loss from the working electrode when scanning at higher negative potentials. Bulky organic ligands such as OLA and TOP impede the electrical conductivity and obstruct active surface sites resulting in a lower HER activity. Hence, the working electrodes were annealed at 450 °C for 2 h under a 5% H_2_/Ar atmosphere to remove organic surface functionalities while maintaining the hexagonal Ni_2_P structure and composition (Supporting Information, Figure S10). Annealing also improves the ohmic contact between the Ti foil and Ni_2–*x*_Zn_*x*_P NCs. The FT-IR spectra of annealed samples confirm the removal of OLA/TOP ligands (Supporting Information, Figure S10), which was evident from the loss of C–H stretches at 2938 and 2859 cm^–1^, C=C stretch at 1650 cm^–1^, C–N stretch at 1468 cm^–1^, and the C–P stretch at 1068 cm^–1^.

Nafion-coated Ti foil electrodes showed a negligible HER activity in 1 M KOH and displayed the highest overpotentials (η_–1_ = 440.7 mV). In contrast, binary Ni_2_P showed significantly higher HER activity, producing η_–1_ and η_–10_ at 110.1 ± 2.1 and 216.2 ± 4.4 mV, respectively, which are lower than those reported for phase pure Ni NCs^53^ and consistent with literature reports of Ni_2_P NCs.^28^

**Figure 5:**
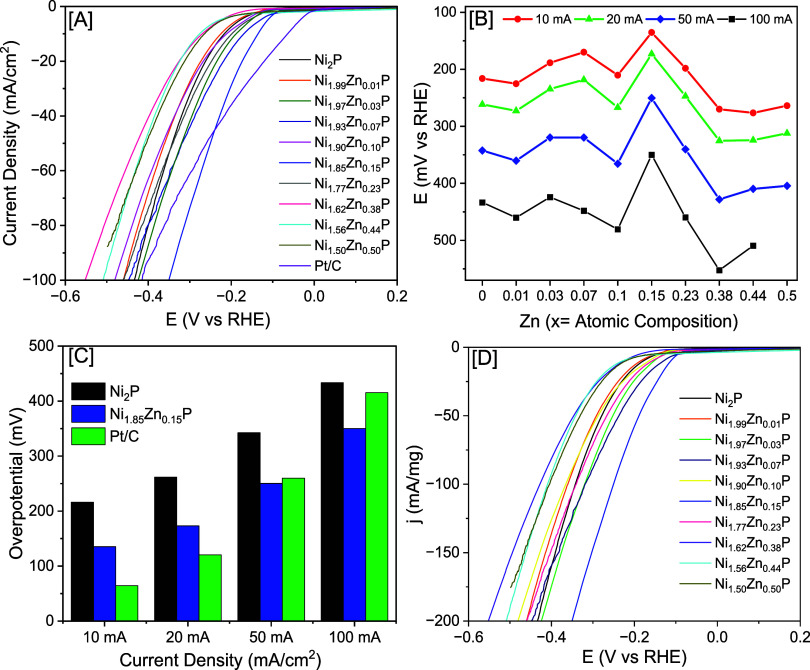
[A] Geometrical area-normalized polarization plots of Ni_2–*x*_Zn_*x*_P NCs and commercial Pt/C in N_2_-saturated 1 M KOH at a 5 mV/s scan rate. [B] Variation of overpotential at different current densities (−10, −20, −50, and −100 mA/cm^2^) as a function of Zn content for Ni_2–*x*_Zn_*x*_P NCs. [C] A bar plot diagram showing the comparison of catalytic activity of Ni_2_P, Ni_1.85_Zn_0.15_P, and Pt/C at different current densities. [D] Mass-normalized LSV plots of Ni_2–*x*_Zn_*x*_P NCs in 1 M KOH at a 5 mV/s scan rate.

XPS spectra of Ni_2–*x*_Zn_*x*_P NCs suggest a shift in Ni^δ+^ and the P^δ−^ peaks to higher binding energies with increasing Zn composition. However, the HER activity was not observed to increase linearly with Zn concentration and displayed an oscillating effect on the Zn composition. Specifically, when the Zn content in Ni_2–*x*_Zn_*x*_P NCs is minimal (*x* = 0.01 equiv to 0.41%) or significantly higher, *x* = 0.38 (13.23%), 0.44 (15.32%), and 0.50 (17.14%), the Ni_2–*x*_Zn_*x*_P NCs showed an η_–10_ of 225.3 ± 3.2, 269.9 ± 4.3, 276.4 ± 3.7, and 263.9 ± 4.9 mV, respectively, which are higher than binary Ni_2_P (216.2 ± 4.4 mV) suggesting a notable decrease in HER activity (Figure 5). However, Ni_2–*x*_Zn_*x*_P NCs with *x* = 0.03 (1.26%), 0.07 (2.60%), 0.10 (3.69%), 0.15 (5.24%), and 0.23 (8.17%) compositions showed an η_–10_ of 188.7 ± 3.7, 170.0 ± 4.2, 210.4 ± 2.8, 135.4 ± 3.4, and 198.3 ± 2.1 mV, respectively, suggesting a higher catalytic activity than binary Ni_2_P. Interestingly, Ni_1.85_Zn_0.15_P NCs showed the lowest η_–10_ of 135.4 ± 3.4 mV among all samples investigated. To understand the catalytic behavior of Ni_2–*x*_Zn_*x*_P NCs with increasing current density, the η values were probed at *j* = −20, −50, and −100 mA/cm^2^. All samples displayed a similar composition-dependent trend observed at *j* = −10 mA/cm^2^ (Figure 5 and Table 2). The HER activities of Ni_2–*x*_Zn_*x*_P NCs were compared with the commercial Pt/C catalyst. Pt/C requires an η_–10_ of 64.3 ± 2.1 mV, which is lower than the highest performing Ni_1.85_Zn_0.15_P NCs. However, at higher current densities, the difference between the η (Pt/C vs Ni_1.85_Zn_0.15_P NCs) becomes minimal, and when *j* ≥−45.1 mA/cm^2^, the Ni_1.85_Zn_0.15_P NCs outperformed the HER activity of Pt/C. Specifically, Pt/C showed η_–50_ and η_–100_ of 259.9 ± 5.8 and 415.3 ± 6.1 mV, while Ni_1.85_Zn_0.15_P NCs showed η_–50_ and η_–100_ of 250.4 ± 5.1 and 350.1 ± 6.2 mV, respectively. The enhanced HER activity demonstrated by Ni_1.85_Zn_0.15_P NCs at higher *j* values is crucial for the development of efficient HER catalysts for industrial applications.

**Table 2:** Comparison of Overpotentials, Tafel Slopes, and ECSAs of Ni_2–*x*_Zn_*x*_P Nanocatalysts

	overpotentials (mV) at different current density^a^					
sample name	–10 mA/cm^2^	–20 mA/cm^2^	–50 mA/cm^2^	–100 mA/cm^2^	Tafel slope (mV/dec)^a^^a^	ECSA (cm^2^)
Ni_2_P	216.2 ± 4.4	261.5 ± 4.8	342.4 ± 6.0	433.4 ± 8.4	111.3 ± 1.2	0.172
Ni_1.99_Zn_0.01_P	225.3 ± 3.2	272.9 ± 5.4	360.3 ± 6.3	460.1 ± 7.8	128.6 ± 0.4	0.036
Ni_1.97_Zn_0.03_P	188.7 ± 3.7	234.5 ± 4.0	319.7 ± 7.1	424.2 ± 9.1	99.7 ± 1.1	0.146
Ni_1.93_Zn_0.07_P	170.0 ± 4.2	218.4 ± 5.8	319.7 ± 6.7	448.1 ± 9.3	134.1 ± 1.0	0.394
Ni_1.90_Zn_0.10_P	210.4 ± 2.8	267.0 ± 5.3	365.5 ± 7.6	480.7 ± 6.7	95.4 ± 1.1	0.096
Ni_1.85_Zn_0.15_P	135.4 ± 3.4	173.2 ± 4.9	250.4 ± 5.1	350.1 ± 6.2	72.1 ± 1.0	0.402
Ni_1.77_Zn_0.23_P	198.3 ± 2.1	247.2 ± 5.2	340.4 ± 5.9	459.8 ± 7.9	133.4 ± 0.9	0.273
Ni_1.62_Zn_0.38_P	269.9 ± 4.3	325.3 ± 5.6	428.2 ± 8.6	552.4 ± 9.6	135.7 ± 0.7	0.130
Ni_1.56_Zn_0.44_P	276.4 ± 3.7	324.3 ± 6.9	409.7 ± 7.8	509.5 ± 9.8	157.2 ± 0.5	0.342
Ni_1.50_Zn_0.5_P	263.9 ± 4.9	312.3 ± 6.1	404.4 ± 8.2	*X*	145.9 ± 0.3	0.381
Pt/C	64.3 ± 2.1	120.4 ± 4.3	259.9 ± 5.8	415.3 ± 6.1	68.7 ± 1.1	*X*

a Overpotentials and Tafel slopes were calculated by employing the LSV curves of three individually prepared electrodes, and the average values are presented. ECSA values were calculated by measuring the double layer capacitance (*C*_DL_) of Ni_2–*x*_Zn_*x*_P catalysts.

The HER activity is also known to vary based on the mass loading of the catalyst.^54^ Therefore, mass-normalized current density was calculated to eliminate any influence from mass loading. The highest performing Ni_1.85_Zn_0.15_P NCs required η of 112.4, 251.3, and 348.8 mV to reach *j* = −10, −100, and −200 mA/mg, respectively, which are lower than other Zn-doped Ni_2_P catalysts (Figure 5D). The ECSAs of Ni_2–*x*_Zn_*x*_P NCs were calculated to investigate the surface specific activity of all samples. ECSA was determined by calculating double layer capacitance (*C*_DL_), derived from CVs scanned in the non-Faradaic regions at different scan rates. The computed ECSAs of Ni_2–*x*_Zn_*x*_P NCs varied from 0.036 to 0.402 cm^2^ without any correlation with the Zn composition (Supporting Information, Figure S11).

**Figure 6:**
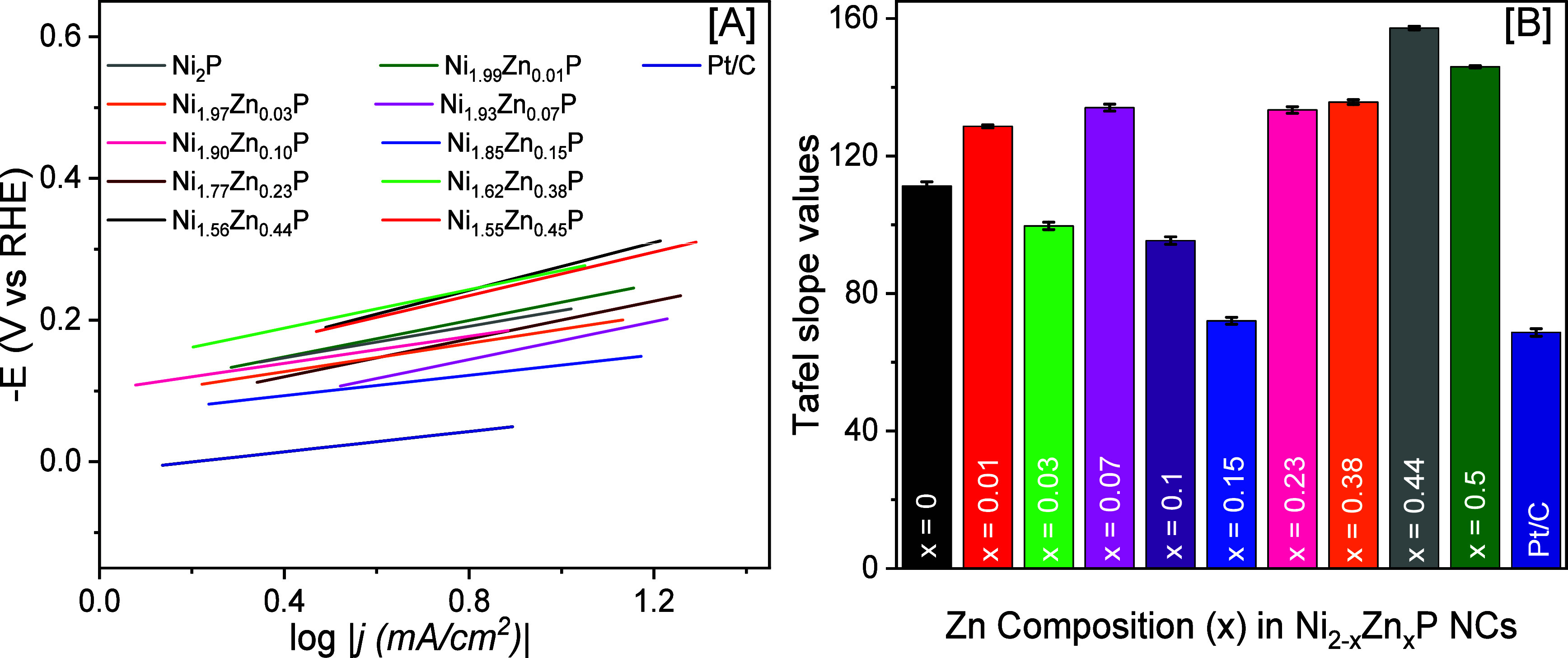
[A] Tafel plots derived from polarization curves of Ni_2–*x*_Zn_*x*_P and commercial Pt/C in N_2_-saturated 1 M KOH at a scan rate of 5 mV/s and [B] a bar plot diagram demonstrating the variation of Tafel slopes as a function of Zn composition and Pt/C.

To further understand the effect of Zn doping on the reaction kinetics and mechanisms, Tafel slopes were computed from the LSV plots. Commercial Pt/C and Ni_2–*x*_Zn_*x*_P NCs produced Tafel slopes of 68.7 ± 1.1 to 157.2 ± 0.5 mV/dec, suggesting Volmer–Heyrovsky mixed HER mechanism (Figure 6 and Table 2).^42,43,45^ Binary Ni_2_P displayed a Tafel slope of 111.3 ± 1.2 mV/dec, whereas Ni_2–*x*_Zn_*x*_P NCs with *x* = 0.01, 0.23, 0.38, 0.44, and 0.50 showed Tafel slopes of 128.6 ± 0.4, 133.4 ± 0.9, 135.7 ± 0.7, 157.2 ± 0.5, and 145.9 ± 0.3 mV/dec, respectively. These values suggest that the Volmer water adsorption (H_2_O + e^–^ → H* + OH^–^) step is the rate-determining step. When Zn content was increased from *x* = 0.03 to 0.15, a decrease in the Tafel slope was noted. Ni_1.97_Zn_0.03_P, Ni_1.90_Zn_0.10_P, and Ni_1.85_Zn_0.15_P NCs produced Tafel slopes of 99.7 ± 1.1, 95.4 ± 1.1, and 72.1 ± 1.0 mV/dec, respectively, suggesting a Heyrovsky rate-determining step (H_2_O + H* + e^–^ → H_2_ + OH^–^). The Tafel slope of the highest active Ni_1.85_Zn_0.15_P NCs (72.1 ± 1.0 mV/dec) was closer to that of Pt/C (68.7 ± 1.1 mV/dec), suggesting similar HER kinetics for both catalysts.

DFT studies indicate that introducing Zn into Ni_2_P brings the Δ*G*_H_ closer to a thermoneutral value, |Δ*G*_H_| ∼0 eV, that can improve the HER activity. Therefore, the dependence of Δ*G*_H_ on Zn composition was also investigated to understand the high activity of the best-performing Ni_1.85_Zn_0.15_P NCs and composition-dependent trends in Δ*G*_H_. Specifically, at a Zn concentration of 2.78%, the first Δ*G*_H_ remained unchanged at −0.42 eV, with hydrogen binding at the Ni_3_-hollow site, similar to pristine Ni_2_P. Meanwhile, the second Δ*G*_H_ increased from 0.15 to −0.13 eV, with hydrogen binding at the Ni–Ni bridge. The overall Δ*G*_H_ binding, represented as |Δ*G*_H1_| + |Δ*G*_H2_|, only decreased slightly from 0.57 eV in pristine Ni_2_P to 0.55 eV in Ni_2_P with 2.78% Zn. At a Zn concentration of 5.56%, the first and second Δ*G*_H_, with hydrogen binding at the Ni–Ni bridge and Ni–P bridge sites, resulted in a net Δ*G*_H_ binding of 0.43 eV. With 11.11% Zn, the first and second Δ*G*_H_ bindings decreased to 0.23 and 0.50 eV, respectively, producing a net Δ*G*_H_ binding of 0.73 eV. These adsorptions occur at the Ni–Zn bridge and Zn–P bridge sites, respectively. The computational models for the hydrogen binding sites on Zn-doped Ni_2_P at variable Zn compositions are shown in Supporting Information, Figure S12. A smaller net Δ*G*_H_ binding suggests higher HER performance, indicating that Ni_2_P with 5.56% Zn should exhibit the highest electrocatalytic activity. These findings are consistent with experimental observations, where a 5.24% Zn composition, corresponding to Ni_1.85_Zn_0.15_P NCs, demonstrated the highest HER activity.

Chronopotentiometry was employed to investigate the effects of Zn doping on the stability and durability of Ni_2–*x*_Zn_*x*_P NCs. The stability of binary Ni_2_P and the highest active Ni_1.85_Zn_0.15_P NCs were investigated at *j* = −10 mA/cm^2^ for 10 h and compared with the Pt/C catalyst (10% wt.). Ni_2_P and Ni_1.85_Zn_0.15_P NCs exhibit similar electrochemical stability in 1 M KOH and showed a modest increase in η_–10_ to 274.1 and 172.9 mV (corresponding to a 26.78 and 27.69% increase), respectively. In contrast, Pt/C displayed a notable increase in η_–10_ to 87.8 mV (36.55% increase) after 10 h of HER. Physical characterization of Ni_2–*x*_Zn_*x*_P NCs after catalysis suggests that the morphology and crystal structure are retained, although a few minor peaks corresponding to tetragonal Ni_12_P_5_ were observed for certain samples (Supporting Information, Figures S13 and S14).

**Figure 7:**
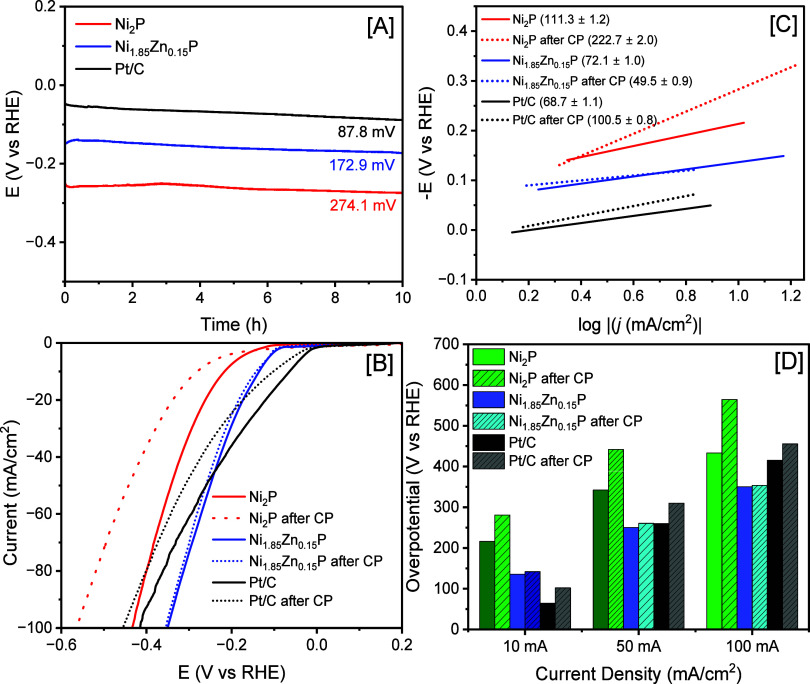
[A] Chronopotentiometry study of Ni_2_P, Ni_1.85_Zn_0.15_P, and commercial Pt/C electrocatalysts at *j* = −10 mA/cm^2^ for 10 h in N_2_-saturated 1 M KOH. [B] Polarization curves, [C] corresponding Tafel slopes, and [D] a bar plot diagram showing the variation of HER overpotentials before (solid line) and after (dotted line) the stability test.

To compare the electrocatalytic activity before and after the chronopotentiometry study, polarization data were recorded and are shown in Figure 7B,D. The LSV data suggest that, after the durability test, Ni_2_P showed a significant decrease in the HER activity characterized by an increase of η_–10_ from 216.2 to 280.9 mV, η_–50_ from 342.4 to 441.9 mV, and η_–100_ from 433.4 to 564.6 mV, corresponding to an overall ∼30% increase from initial η values. In contrast, commercial Pt/C showed an increase of η_–10_ from 64.3 to 102.1 mV (58.79% increase), an increase of η_–50_ from 259.9 to 310.0 mV (19.27% increase), and an increase of η_–100_ from 415.3 to 455.5 mV (9.68% increase). The highest performing Ni_1,85_Zn_0.15_P NCs demonstrated excellent stability in alkaline electrolytes with a negligible change in η after 10 h of continuous HER. For Ni_1.85_Zn_0.15_P NCs, the η_–10_ changed from 135.4 to 142.0 (4.87% increase), the η_–50_ changed from 250.4 to 260.5 mV (4.03% increase), and the η_–100_ changed from 350.1 to 353.6 mV (0.99% increase). LSV plots recorded after the stability test were used to calculate Tafel slopes. An obvious increase in the Tafel slope from 111.3 ± 1.2 to 222.7 ± 2.0 mV/dec (100.08% increase) was observed for binary Ni_2_P, whereas the slope for Pt/C increased from 68.7 ± 1.1 to 100.5 ± 0.8 mV/dec, corresponding to a 46.28% increase. These increases in slope values indicate that, after the stability test, Ni_2_P and Pt/C favor the Volmer HER mechanism, attributing to slower HER kinetics and/or decreased electrocatalytic efficiency. In contrast, Ni_1.85_Zn_0.15_P NCs demonstrated a decrease in the Tafel slope from 72.1 ± 1.0 to 49.5 ± 0.9 (31.35% decrease), suggesting more dominant Heyrovsky type reaction with enhanced HER kinetics. This suggests that, with appropriate Zn doping, both the HER activity and stability of hexagonal Ni_2_P can be greatly improved.

## Conclusions

In this work, theoretical and experimental methods were integrated to identify the most promising HER dopant and dopant concentration for hexagonal Ni_2_P and the HER activity and stability of Ni_2–*x*_Zn_*x*_P NCs were systematically probed as a function of Zn composition. DFT studies suggest that the Zn composition has a direct effect on the Δ*G*_H_ of Ni_2–*x*_Zn_*x*_P NCs where 5.56% Zn is predicted to lower the Δ*G*_H_ more favorably than 2.78 and 11.11% Zn. Accordingly, hexagonal Ni_2–*x*_Zn_*x*_P NCs with variable Zn compositions (*x* = 0–0.50) were synthesized via a colloidal route and homogeneous alloy formation with no evidence of Zn segregation was confirmed through STEM-EDS. XPS spectra indicate modulation of surface polarization due to changes in binding energies of Ni and P moieties upon Zn incorporation into Ni_2_P NCs. At low and high Zn compositions, a decrease in HER activity was noted as *x* = 0.01, 0.38, 0.44, and 0.50 compositions exhibit η_–10_ of 225.3 ± 3.2, 269.9 ± 4.3, 276.4 ± 3.7, and 263.9 ± 4.9 mV, respectively, which are higher than binary Ni_2_P NCs (216.9 mV). The lowest η_–10_ of 135.4 ± 3.4 mV was achieved for Ni_1.85_Zn_0.15_P NCs (5.24% Zn) consistent with DFT studies, which predicted the optimum HER activity at ∼5.56% Zn composition. Moreover, Ni_1.85_Zn_0.15_P NCs outperformed the HER activity of Pt/C (10% wt.) standard at *j* ≥−45.1 mA/cm^2^ with an η_–50_ of 250.4 ± 5.1 mV compared to an η_–50_ of 259.9 ± 5.8 mV achieved for Pt/C. A comparison of polarization curves recorded at various *j* values before and after 10 h of HER indicates that Ni_1.85_Zn_0.15_P NCs display the highest corrosion tolerance and retain its high activity for a longer duration compared to benchmark Pt/C. More specifically, Ni_2_P NCs displayed an increase in η_–10_ from 216.2 to 280.9 mV, whereas Ni_1.85_Zn_0.15_P NCs and Pt/C showed increases from 135.4 to 142.0 mV and 64.3 to 102.1 mV, respectively. In summary, the dopant identity and concentration are critical parameters when designing an efficient electrocatalyst. When these considerations are optimized, the catalytic activity and stability can be improved to such an extent that it can rival expensive noble metal catalysts. This study continues the inquisition into the optimization of earth-abundant catalysts for water electrolysis reaction and demonstrates how doping can be employed to modulate the efficiency and stability of catalytic nanostructures.

## Supporting Information Available

A diagram demonstrating the lowest first and second Δ*G*_H_ values on pristine Ni_2_P (001) with the Ni_3_P_2_ termination along with corresponding 9 transition metal-doped surfaces; size histograms of Ni_2–*x*_Zn_*x*_P NCs with variable compositions; LRTEM images of Ni_2–*x*_Zn_*x*_P NCs with *x* = 0.03, 0.07, and 0.10 compositions; STEM images and elemental maps of Ni_2–*x*_Zn_*x*_P NCs with *x* = 0.23, *x* = 0.15, and 0.50 compositions; XPS spectra of Ni_2_P NCs; FT-IR spectra of Ni_2_P NCs after annealing at 450 °C for 2 h; PXRD patterns of Ni_2–*x*_Zn_*x*_P NCs after annealing at 450 °C for 2 h; CV curves and corresponding ECSA plots of Ni_2_P and Ni_1.85_Zn_0.15_P NCs; the lowest first and second Δ*G*_H_ on Zn-doped Ni_2_P (001) with the Ni_3_P_2_ termination at Zn concentrations of 2.78, 5.56, and 11.11%; and LRTEM and PXRD pattern of Ni_1.90_Zn_0.10_P NCs recorded after HER (PDF)

## References

[ref1] Joo J.; Kim T.; Lee J.; Choi S.; Lee K. Morphology-Controlled Metal Sulfides and Phosphides for Electrochemical Water Splitting. Adv. Mater. 2019, 31 (14), 1806682 10.1002/adma.201806682. 30706578

[ref2] Wang Y.; Kong B.; Zhao D.; Wang H.; Selomulya C. Strategies for Developing Transition Metal Phosphides as Heterogenous Electrocatalysts for Water Splitting. Nano Today 2017, 15, 26–55. 10.1016/j.nantod.2017.06.006.

[ref3] Ray A.; Sultana S.; Paramanik L.; Parida K. M. Recent Advances in Phase, Size, and Morphology-Oriented Nanostructured Nickel Phosphide for Overall Water Splitting. J. Mater. Chem. A 2020, 8, 19196–19245. 10.1039/D0TA05797E.

[ref4] Jaksic J.; Ristic N. M.; Krstajic N. V.; Jaksic M. M. Electrocatalysis for Hydrogen Electrode Reactions in the Light of Fermi Dynamics and Structural Bonding FACTORS-I.. Individual Electrocatalytic Properties of Transition Metals. Int. J. Hydrogen Energy 1998, 23 (12), 1121–1156. 10.1016/S0360-3199(98)00014-7.

[ref5] Li Y.; Zhang X.; Liu L.; Sheng H.; Li C.; Cao L.; Li H.; Xia C.; Dong B. Ultra-Low Pt Doping and Pt-Ni Pair Sites in Amorphous/Crystalline Interfacial Electrocatalyst Enable Efficient Alkaline Hydrogen Evolution. Small 2023, 19 (23), 2300368 10.1002/smll.202300368. 36879475

[ref6] Li Y.; Wu Z.; Zhang X.; Song F.; Cao L.; Sheng H.; Gao X.; Li C.; Li H.; Li W.; Dong B. Interfacial Engineering of Polycrystalline Pt_5_P_2_ Nanocrystals and Amorphous Nickel Phosphate Nanorods for Electrocatalytic Alkaline Hydrogen Evolution. Small 2023, 19 (9), 2206859 10.1002/smll.202206859. 36564350

[ref7] Zeng B.; Liu X.; Wan L.; Xia C.; Cao L.; Hu Y.; Dong B. Grafting Ultra-Fine Nanoalloys with Amorphous Skin Enables Highly Active and Long-Lived Acidic Hydrogen Production. Angew. Chem. Int. Ed. 2024, 63 (15), e202400582 10.1002/anie.202400582. 38308672

[ref8] Balamurugan J.; Nguyen T. T.; Kim D. H.; Kim N. H.; Lee J. H. 3D Nickel Molybdenum Oxyselenide (Ni_1-x_Mo_x_OSe) Nanoarchitecture as Advanced Multifunctional Catalyst for Zn-Air Batteries and Water Splitting. Appl. Catal., B 2021, 286, 119909 10.1016/j.apcatb.2021.119909.

[ref9] Balamurugan J.; Nguyen T. T.; Aravindan V.; Kim N. H.; Lee J. H. Highly Reversible Water Splitting Cell Building From Hierarchical 3D Nickel Manganese Oxyphosphide Nanoheets. Nano Energy 2020, 69, 104432 10.1016/j.nanoen.2019.104432.

[ref10] Balamurugan J.; Austeria P. M.; Kim J. B.; Jeong E.-S.; Huang H.-H.; Kim D. H.; Koratkar N.; Kim S. O. Electrocatalysts for Zinc-Air Batteries Featuring Single Molybdenum Atoms in a Nitrogen-Doped Carbon Framework. Adv. Mater. 2023, 35 (35), 2302625 10.1002/adma.202302625. 37327064

[ref11] Hernandez A. B.; Ariga H.; Takakusagi S.; Kinoshita K.; Suzuki S.; Otani S.; Oyama S. T.; Asakura K. Dynamical LEED Analysis of Ni_2_P (0001)-1 × 1: Evidence for P-Covered Surface Structure. Chem. Phys. Lett. 2011, 513 (1), 48–52. 10.1016/j.cplett.2011.07.055.

[ref12] Liu P.; Rodriguez J. A. Catalysts for Hydrogen Evolution from the [NiFe] Hydrogenase to the Ni_2_P (001) Surface. J. Am. Chem. Soc. 2005, 127 (42), 14871–14878. 10.1021/ja0540019. 16231942

[ref13] Popczun E. J.; McKone J. R.; Read C. G.; Biacchi A. J.; Wiltrout A. M.; Lewis N. S.; Schaak R. E. Nanostructured Nickel Phosphide as an Electrocatalyst for the Hydrogen Evolution Reaction. J. Am. Chem. Soc. 2013, 135, 9267–9270. 10.1021/ja403440e. 23763295

[ref14] Kibsgaard J.; Tsai C.; Chan K.; Benck J. D.; Nørskov J. K.; Abild-Pedersen F.; Jaramillo T. F. Designing an Improved Transition Metal Phosphide Catalyst for Hydrogen Evolution Using Experimental and Theoretical Trends. Energy Environ. Sci. 2015, 8, 3022–3029. 10.1039/C5EE02179K.

[ref15] Sun H.; Min Y.; Yang W.; Lian Y.; Lin L.; Feng K.; Deng Z.; Chen M.; Zhong J.; Xu L.; Peng Y. Morphological and Electronic Tuning of Ni_2_P Through Iron Doping Toward Highly Efficient Water Splitting. ACS Catal. 2019, 9 (10), 8882–8892. 10.1021/acscatal.9b02264.

[ref16] Hakala M.; Laasonen K. Hydrogen Adsorption Trends on Al-Doped Ni_2_P Surfaces for Optimal Catalyst Design. Phys. Chem. Chem. Phys. 2018, 20, 13785–13791. 10.1039/C8CP00927A. 29744495

[ref17] Partanen L.; Hakala M.; Laasonen K. Hydrogen Adsorption Trends on Various Metal-Doped Ni_2_P Surfaces for Optimal Catalysts Design. Phys. Chem. Chem. Phys. 2019, 21, 184–191. 10.1039/C8CP06143B. 30516185

[ref18] Partanen L.; Alberti S.; Laasonen K. Hydrogen Adsorption Trends on Two Metal-Doped Ni_2_P Surfaces for Optimal Catalyst Design. Phys. Chem. Chem. Phys. 2021, 23, 11538–11547. 10.1039/D1CP00684C. 33969865

[ref19] Fan S.; Zhang J.; Wu Q.; Huang S.; Zheng J.; Kong D.; Chen S.; Wang Y.; Ang L. K.; Shi Y.; Yang H. Y. Morphological and Electronic Dual Regulation of Cobalt-Nickel Bimetal Phosphide Heterostructures Inducing High Water-Splitting Performance. J. Phys. Chem. Lett. 2020, 11, 3911–3919. 10.1021/acs.jpclett.0c00851. 32320249

[ref20] Li Y.; Yu X.; Gao J.; Ma Y. Hierarchical Ni_2_P/Zn-Ni-P Nanosheet Array for Efficient Energy-Saving Hydrogen Evolution and Hydrazine Oxidation. J. Mater. Chem. A 2023, 11 (5), 2191–2202. 10.1039/D2TA08366C.

[ref21] Wen L.; Yu J.; Xing C.; Liu D.; Lyu X.; Cai W.; Li X. Flexible Vanadium-Doped Ni_2_P Nanosheet Arrays Grown on Carbon Cloth for an Efficient Hydrogen Evolution Reaction. Nanoscale 2019, 11, 4198–4203. 10.1039/C8NR10167A. 30806413

[ref22] Nørskov J. K.; Bligaard T.; Loagdottir A.; Kitchin J. R.; Chen J. G.; Pandelov S.; Stimming U. Trends in the Exchange Current for Hydrogen Evolution. J. Electrochem. Soc. 2005, 152 (3), J23–J26. 10.1149/1.1856988.

[ref23] Jin Z.; Li P.; Huang X.; Zeng G.; Jin Y.; Zheng B.; Xiao D. Three-Dimensional Amorphous Tungsten-Doped Nickel Phosphide Microsphere as an Efficient Electrocatalysts for Hydrogen Evolution. J. Mater. Chem. A 2014, 2, 18593–18599. 10.1039/C4TA04434G.

[ref24] Lado J. L.; Wang X.; Paz E.; Carbó-Argibay E.; Guldris N.; Rodríguez-Abreu C.; Liu L.; Kovnir K.; Kolen’ko Y. V. Design and Synthesis of Highly Active Al-Ni-P Foam Electrode for Hydrogen Evolution Reaction. ACS Catal. 2015, 5, 6503–6508. 10.1021/acscatal.5b01761.

[ref25] Sun Y.; Hang L.; Shen Q.; Tao Z.; Li H.; Zhang X.; Lyu X.; Li Y. Mo Doped Ni_2_P Nanowire Arrays: An Efficient Electrocatalyst for the Hydrogen Evolution Reaction with Enhanced Activity at All pH Values. Nanoscale 2017, 9, 16674–16679. 10.1039/C7NR03515B. 28820219

[ref26] Man H.-W.; Tsang C.-S.; Li M. M.-J.; Mo J.; Huang B.; Lee L. Y. S.; Leung Y.; Wong K.-Y.; Tsang S. C. E. Transition Metal-Doped Nickel Phosphide Nanoparticles as Electro-and Photocatalysts for Hydrogen Generation Reactions. Appl. Catal., B 2019, 242, 186–193. 10.1016/j.apcatb.2018.09.103.

[ref27] Zhang Y.; Liu Y.; Ma M.; Ren X.; Liu Z.; Du G.; Asiri A. M.; Sun X. A Mn-Doped Ni_2_P Nanosheet Array: An Efficient and Durable Hydrogen Evolution Reaction Electrocatalyst in Alkaline Media. Chem. Commun. 2017, 53, 11048–11051. 10.1039/C7CC06278H. 28944793

[ref28] Eladgham E. H.; Rodene D. D.; Sarkar R.; Arachchige I. U.; Gupta R. B. Electrocatalytic Activity of Bimetallic Ni-Mo-P Nanocrystals for Hydrogen Evolution Reaction. ACS Appl. Nano Mater. 2020, 3, 8199–8207. 10.1021/acsanm.0c01624.

[ref29] Putri L. K.; Ng B.-J.; Yeo R. Y. Z.; Ong W.-J.; Mohamed A. R.; Chai S.-P. Engineering Nickel Phosphides for Electrocatalytic Hydrogen Evolution: A Doping Perspective. Chem. Eng. J. 2023, 461, 141845 10.1016/j.cej.2023.141845.

[ref30] Li D.; Senevirathne K.; Aquilina L.; Brock S. L. Effect of Synthetic Levers on Nickel Phosphide Nanoparticle Formation: Ni_5_P_4_ and NiP_2_. Inorg. Chem. 2015, 54, 7968–7975. 10.1021/acs.inorgchem.5b01125. 26238550

[ref31] Kresse G.; Furthmüller J. Efficient Iterative Schemes for Ab Initio Total Energy Calculations Using a Plane-Wave Basis Set. Phys. Rev. B 1996, 54, 11169–11186. 10.1103/PhysRevB.54.11169. 9984901

[ref32] Greeley J.; Jaramillo T. F.; Bonde J.; Chorkendorff I.; Nørskov J. K. Computational High-Throughput Screening of Electrocatalytic Materials for Hydrogen Evolution. Nat. Mater. 2006, 5 (11), 909–913. 10.1038/nmat1752. 17041585

[ref33] Zheng Y.; Jiao Y.; Zhu Y.; Li L. H.; Han Y.; Chen Y.; Du A.; Jaroniec M.; Qiao S. Z. Hydrogen Evolution by a Metal-Free Electrocatalyst. Nat. Commun. 2014, 5, 3783 10.1038/ncomms4783. 24769657

[ref34] Hu J.; Zheng S.; Zhao X.; Yao X.; Chen Z. A Theoretical Study on the Surface and Interfacial Properties on Ni_3_P for the Hydrogen Evolution Reaction. J. Mater. Chem. A 2018, 6, 7827–7834. 10.1039/C8TA00437D.

[ref35] Huang A.; Dinh K. N.; Sun X.; Yan Q.; Wang Z. Surface Treated Nickel Phosphide Nanosheet with Oxygen as Highly Efficient Bifunctional Electrocatalysts for Overall Water Splitting. Appl. Surf. Sci. 2019, 496, 143741 10.1016/j.apsusc.2019.143741.

[ref36] Hansen M. H.; Stern L.-A.; Feng L.; Rossmeisl J.; Hu X. Widely Available Active Sites on Ni_2_P for Electrochemical Hydrogen Evolution-Insights from First Principles Calculation. Phys. Chem. Chem. Phys. 2015, 17, 10823–10829. 10.1039/C5CP01065A. 25812670

[ref37] Perdew J. P.; Burke K.; Ernzerhof M. Generalized Gradient Approximation Made Simple. Phys. Rev. Lett. 1996, 77, 3865–3868. 10.1103/PhysRevLett.77.3865. 10062328

[ref38] Grimme S.; Antony J.; Ehrlich S.; Krieg H. A Consistent and Accurate Ab Initio Parametrization of Density Functional Dispersion Correction (DFT-D) for the 94 Elements H-Pu. J. Chem. Phys. 2010, 132, 154104 10.1063/1.3382344. 20423165

[ref39] Grimme S.; Ehrlich S.; Goerigk L. The Effect of the Damping Function in Dispersion Corrected Density Functional Theory. J. Comput. Chem. 2011, 32, 1456–1465. 10.1002/jcc.21759. 21370243

[ref40] Wexler R. B.; Martirez J. M. P.; Rappe A. M. Stable Phosphorus-Enriched (0001) Surfaces of Nickel Phosphides. Chem. Mater. 2016, 28, 5365–5372. 10.1021/acs.chemmater.6b01437.

[ref41] Wexler R. B.; Martirez J. M. P.; Rappe A. M. Active Role of Phosphorus in the Hydrogen Evolving Activity of Nickel Phosphide (0001) Surfaces. ACS Catal. 2017, 7 (11), 7718–7725. 10.1021/acscatal.7b02761.

[ref42] Wang T.; Miao L.; Zheng S.; Qin H.; Cao X.; Yang L.; Jiao L. Interfacial Engineering of Ni_3_N/Mo_2_N Heterojunctions for Urea-Assisted Hydrogen Evolution Reaction. ACS Catal. 2023, 13 (7), 4091–4100. 10.1021/acscatal.3c00113.

[ref43] Ren J. T.; Chen L.; Wang H. Y.; Tian W. W.; Song X. L.; Kong Q. H.; Yuan Z. Y. Synergistic Activation of Crystalline Ni_2_P and Amorphous NiMoO_x_ for Efficient Water Splitting at High Current Densities. ACS Catal. 2023, 13 (14), 9792–9805. 10.1021/acscatal.3c01885.

[ref44] Batugedara T.; Brock S. L. A Little Nickel Goes a Long Way: Ni Incorporation into Rh_2_P for Stable Bifunctional Electrocatalytic Water Splitting in Acidic Media. ACS Mater. Au 2023, 3, 299–309. 10.1021/acsmaterialsau.2c00080. 38090124 PMC10347692

[ref45] McCrory C. C. L.; Jung S.; Peters J. C.; Jaramillo T. F. Benchmarking Heterogeneous Electrocatalysts for the Oxygen Evolution Reaction. J. Am. Chem. Soc. 2013, 135 (45), 16977–16987. 10.1021/ja407115p. 24171402

[ref46] Laursen A. B.; Wexler R. B.; Whitaker M. J.; Izett E. J.; Calvinho K. U. D.; Hwang S.; Rucker R.; Wang H.; Li J.; Garfunkel E.; Greenblatt M.; Rappe A. M.; Dismukes G. C. Climbing the Volcano of Electrocatalytic Activity While Avoiding Catalyst Corrosion: Ni_3_P, a Hydrogen Evolution Electrocatalyst Stable in Both Acid and Alkali. ACS Catal. 2018, 8, 4408–4419. 10.1021/acscatal.7b04466.

[ref47] Pan Y.; Liu Y.; Zhao J.; Yang K.; Liang J.; Liu D.; Hu W.; Liu D.; Liu Y.; Liu C. Monodispersed Nickel Phosphide Nanocrystals with Different Phases: Synthesis, Characterization and Electrocatalytic Properties for Hydrogen Evolution. J. Mater. Chem. A 2015, 3, 1656 10.1039/C4TA04867A.

[ref48] Wang J.; Johnston-Peck A. C.; Tracy J. B. Nickel Phosphide Nanoparticles with Hollow, Solid, and Amorphous Structures. Chem. Mater. 2009, 21, 4462–4467. 10.1021/cm901073k.

[ref49] Muthuswamy E.; Savithra G. H. L.; Brock S. L. Synthetic Levers Enabling Independent Control of Phase, Size, and Morphology in Nickel Phosphide Nanoparticles. ACS Nano 2011, 5 (3), 2402–2411. 10.1021/nn1033357. 21381759

[ref50] Graves L. S.; Sarkar R.; Lao K. U.; Arachchige I. U. Composition-Dependent Electrocatalytic Activity of Zn-Doped Ni_5_P_4_ Nanocrystals for the Hydrogen Evolution Reaction. Chem. Mater. 2023, 35 (17), 6966–6978. 10.1021/acs.chemmater.3c01229.

[ref51] Carenco S.; Lui Z.; Salmeron M. The Birth of Nickel Phosphide Catalysts: Monitoring Phosphorus Insertion into Nickel. ChemCatChem 2017, 9, 2318–2323. 10.1002/cctc.201601526.

[ref52] Ji S.; Li J.; Li J.; Song C.; Wang S.; Wang K.; Hui K. S.; Zha C.; Zheng Y.; Dinh D. A.; Chen S.; Zhang J.; Mai W.; Tang Z.; Shao Z.; Hui K. N. Dynamic Reversible Evolution of Solid Electrolyte Interface in Nonflammable Triethyl Phosphate Electrolyte Enabling Safe and Stable Potassium-Ion Batteries. Adv. Funct. Mater. 2022, 32 (28), 2200771 10.1002/adfm.202200771.

[ref53] Rodene D. D.; Eladgham E. H.; Gupta R. B.; Arachchige I. U.; Tallapally V. Crystal Structure and Composition-Dependent Electrocatalytic Activity of Ni-Mo Nanoalloys for Water Splitting to Produce Hydrogen. ACS Appl. Energy Mater. 2019, 2 (10), 7112–7120. 10.1021/acsaem.9b01043.

[ref54] Niu S.; Li S.; Du Y.; Han X.; Xu P. How to Reliably Report the Overpotential of an Electrocatalysts. ACS Energy Lett. 2020, 5 (4), 1083–1087. 10.1021/acsenergylett.0c00321.

