# Supplementary material for: Structure- and Morphology-Controlled Synthesis of Hexagonal Ni2–xZnxP Nanocrystals and Their Composition-Dependent Electrocatalytic Activity for Hydrogen Evolution Reaction
Source: ACS Appl Energy Mater. 2024 Jul 5;7(14):5679–90. doi: 10.1021/acsaem.4c00539 (PMC11267498; doi:10.1021/acsaem.4c00539)
Supplement: Supplementary file 1 — ae4c00539_si_001.pdf [file ae4c00539_si_001.pdf]

Supporting Information

Structure and Morphology Controlled Synthesis of Hexagonal $Ni_{2-x}Zn_xP$ Nanocrystals and Their Composition-Dependent Electrocatalytic Activity for Hydrogen Evolution Reaction

*Lisa S. Graves,[$] Rajib Sarkar,[$] Jordon Baker, Ka Un Lao, Indika U. Arachchige**

Department of Chemistry, Virginia Commonwealth University, Richmond, Virginia 23284-2006, United States.

**Email: iuarachchige@vcu.edu*

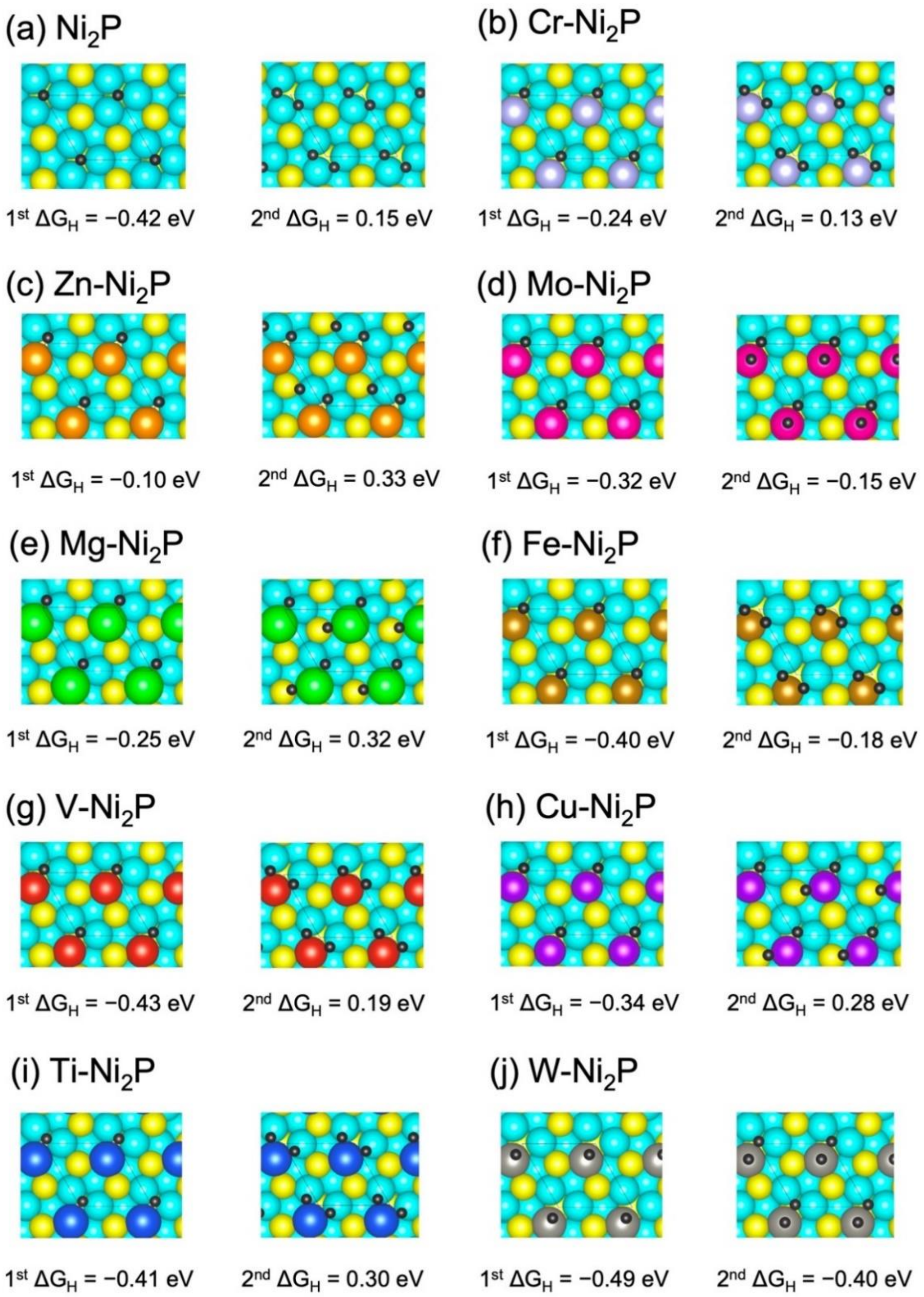

Figure S1. The lowest first and second ΔG_H on pristine (a) Ni_2P (001) with the Ni_3P_2 termination, along with the corresponding 9 transition-metal-doped (b) Cr, (c) Zn, (d) Mo, (e) Mg, (f) Fe, (g) V, (h) Cu, (i) Ti, and (j) W surfaces calculated by PBE+D3(BJ).

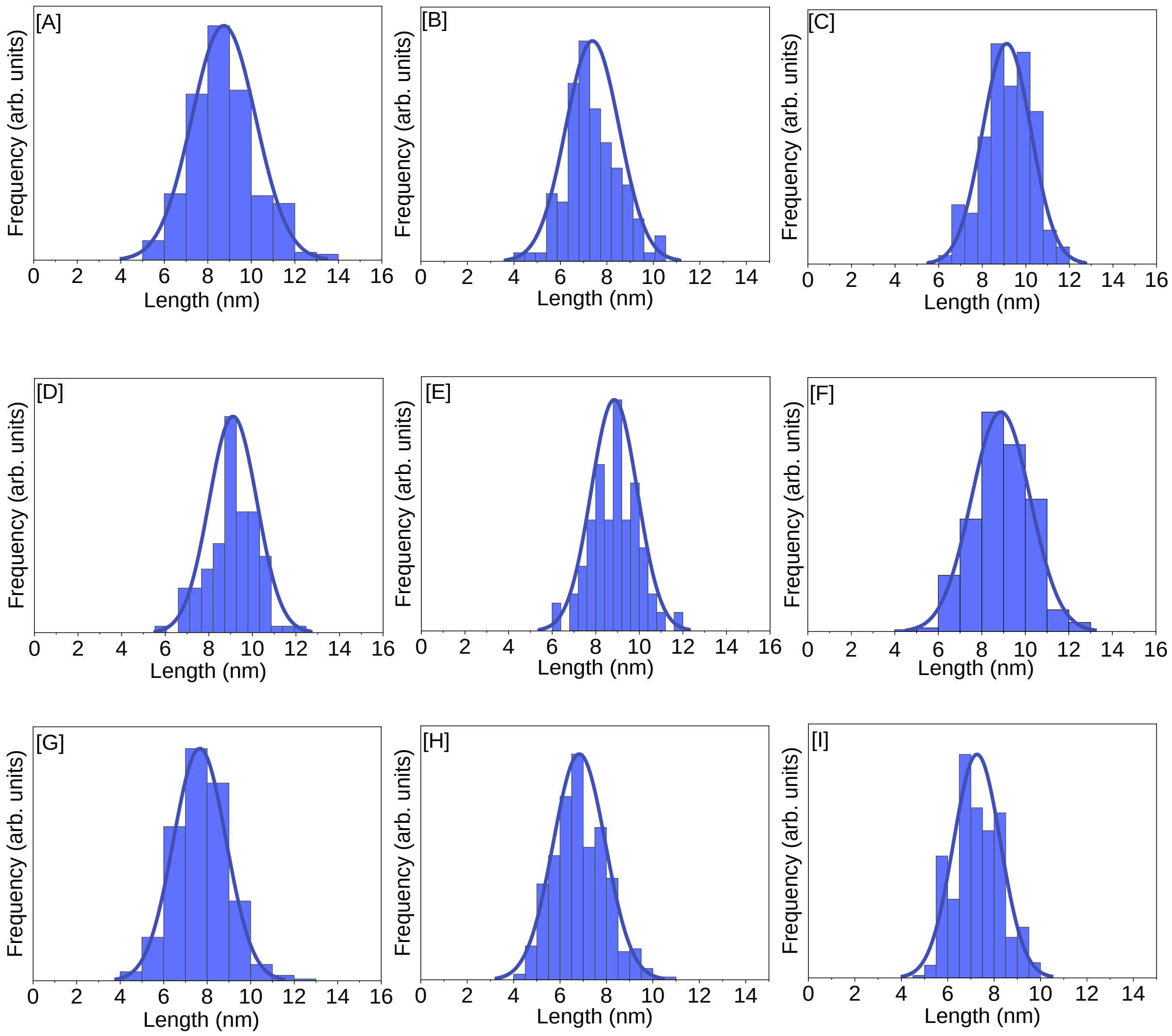

Figure S2. Size histograms of hexagonal $Ni_{2-x}Zn_xP$ NCs with varying Zn compositions: (A) x = 0 (8.7 ± 1.5 nm), (B) x = 0.01 (7.4 ± 1.2 nm), (C) x = 0.03 (9.1 ± 1.1 nm), (D) x= 0.07 (9.1 ± 1.1 nm), (E) x= 0.10 (8.9 ± 1.1 nm), (F) x = 0.15 (8.9 ± 1.4 nm), (G) x = 0.23 (8.5 ± 1.2 nm), (H) x = 0.38 (6.8 ± 1.1 nm), and (I) x = 0.44 (7.3 ± 1.0 nm). The average particle size of each composition is shown in parentheses.

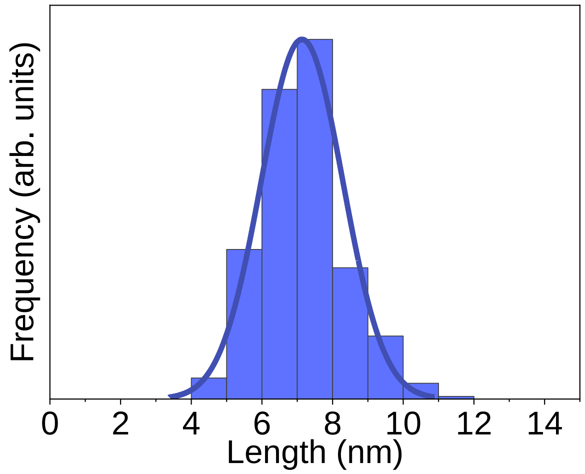

Figure S3. Size histogram of hexagonal $Ni_{1.50}Zn_{0.50}P$ NCs (average particle size = 7.1 ± 1.2 nm).

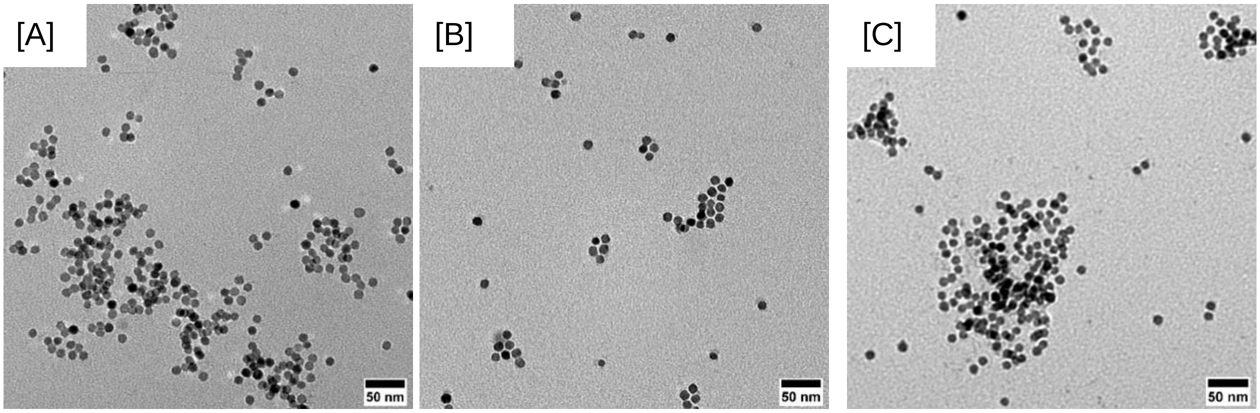

Figure S4. Low resolution TEM images of hexagonal $Ni_{2-x}Zn_xP$ NCs with variable Zn compositions: (A) x = 0.03, (B) x = 0.07 and (C) x = 0.10.

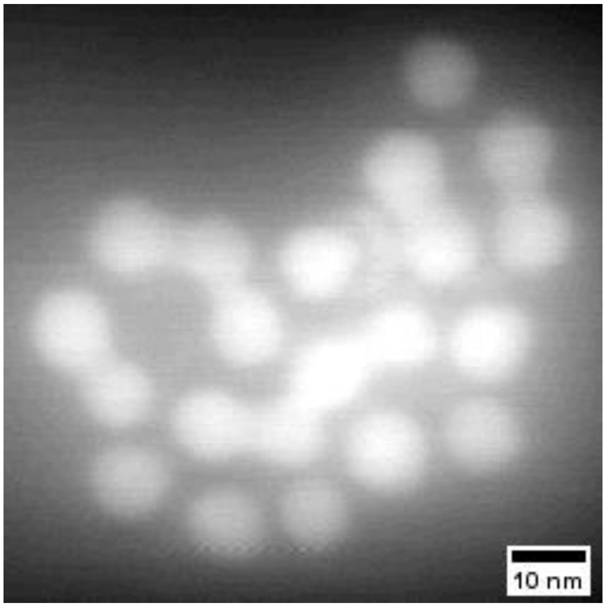

Figure S5. A representative STEM image of $Ni_{1.77}Zn_{0.23}P$ NCs. Corresponding STEM-HAADF Ni, Zn, and P maps are shown in Figure 3 of the main manuscript.

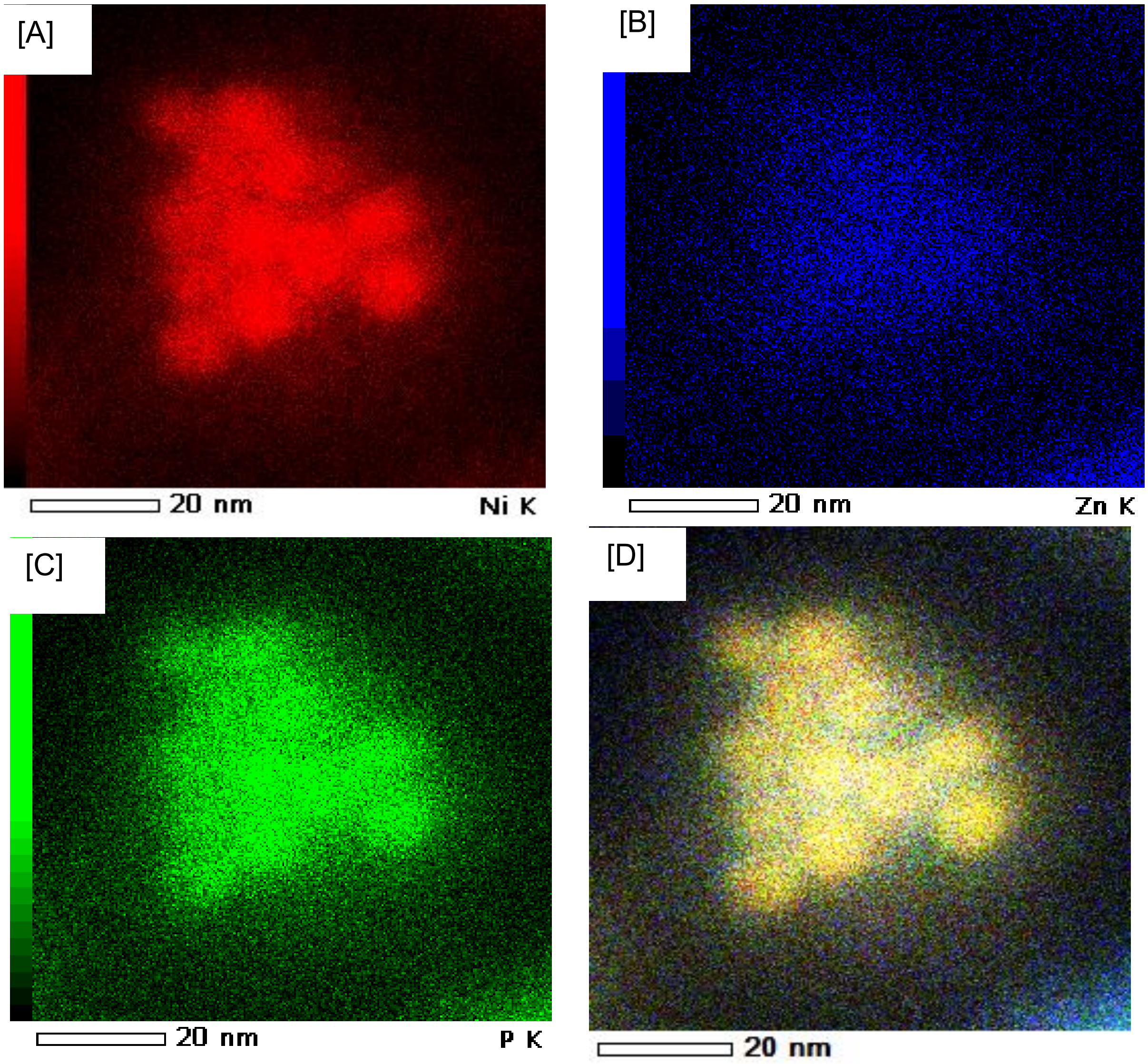

Figure S6. STEM-HAADF elemental maps of (A) Ni, (B) Zn, and (C) P and (D) overlay image obtained for $Ni_{1.50}Zn_{0.50}P$ NCs showing the structural homogeneity of as-synthesized particles.

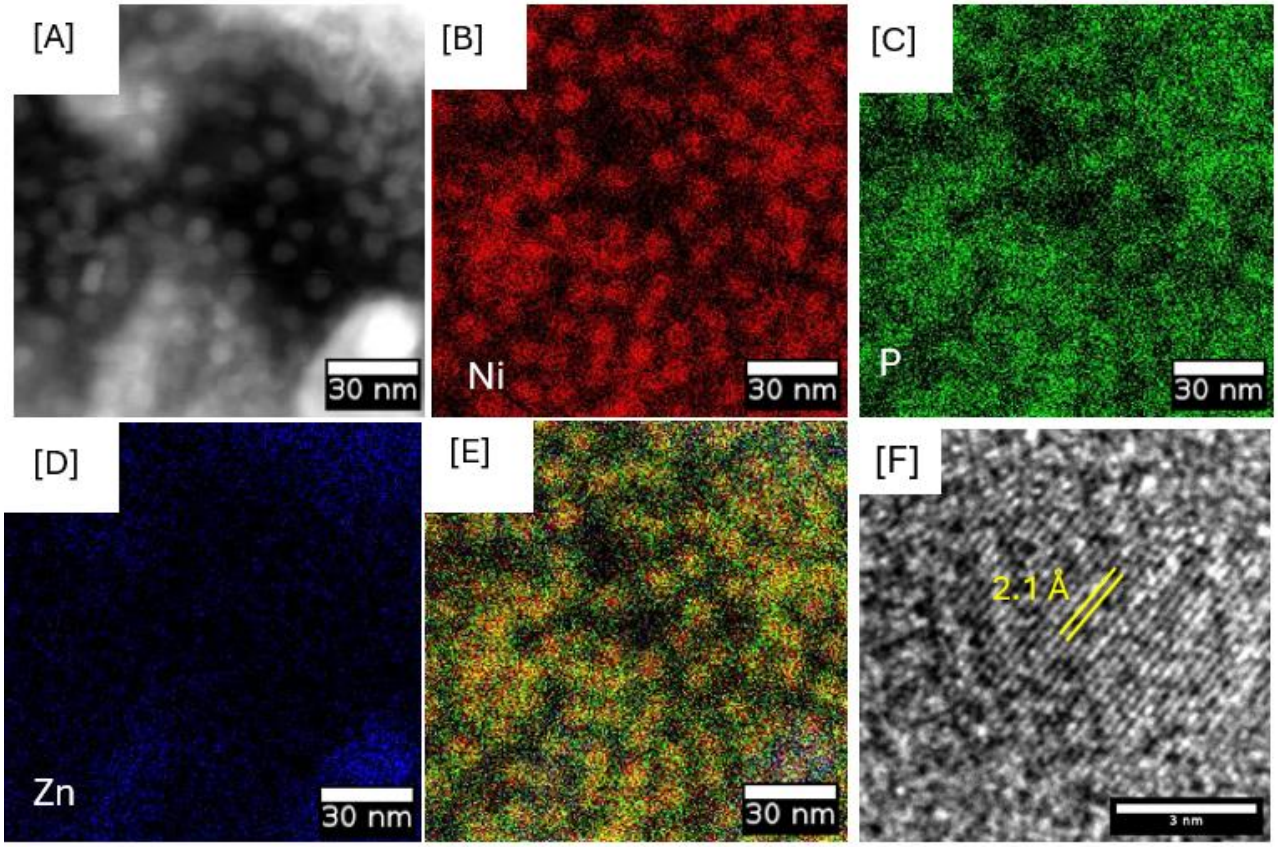

Figure S7. (A) STEM image and STEM-HAADF elemental maps of (B) Ni, (C) P, and (D) Zn and (E) overlay of all elements for $Ni_{1.85}Zn_{0.15}P$ NCs, demonstrating the structural homogeneity of as-synthesized particles. [F] A representative HRTEM image of $Ni_{1.85}Zn_{0.15}P$ NCs.

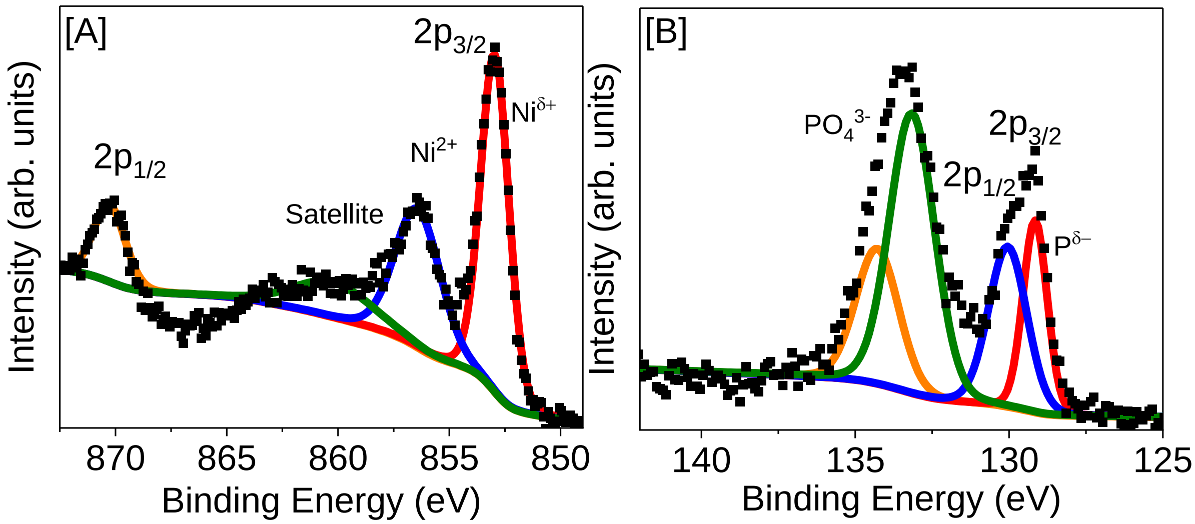

Figure S8. XPS spectra of Ni_2P NCs displaying (A) Ni 2p (B) P 2p regions. Square symbols represent experimental data and colored lines are fitted deconvolutions. Samples were annealed at 450 °C for 2 h under 5% H_2:Ar atmosphere.

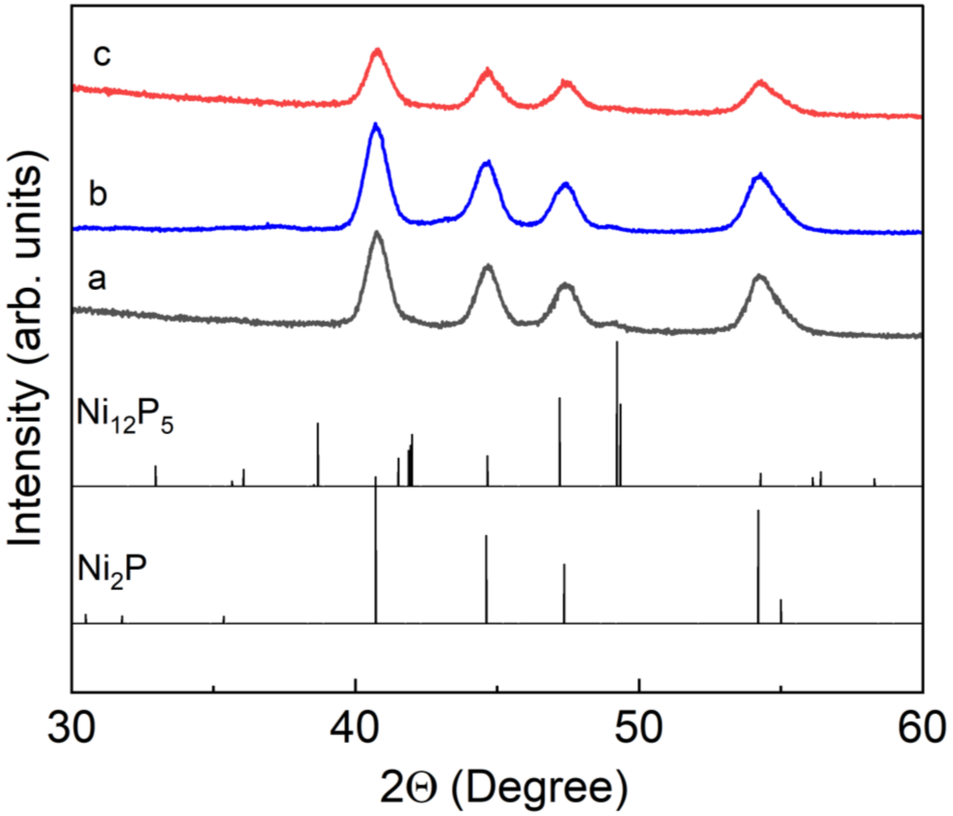

Figure S9. Power X-ray diffraction patterns of Ni_2P and $Ni_{2-x}Zn_xP$ NCs with variable Zn compositions: (a) x = 0, (b) x = 0.10, (c) x = 0.23 after annealing at 450 °C for 2 h under 5% H_2/Ar atmosphere. $Ni_{12}P_5$ (JCPDS No. 04-003-6050) and Ni_2P (JCPDS No. 01-074-1385) reference patterns are shown as black vertical lines.

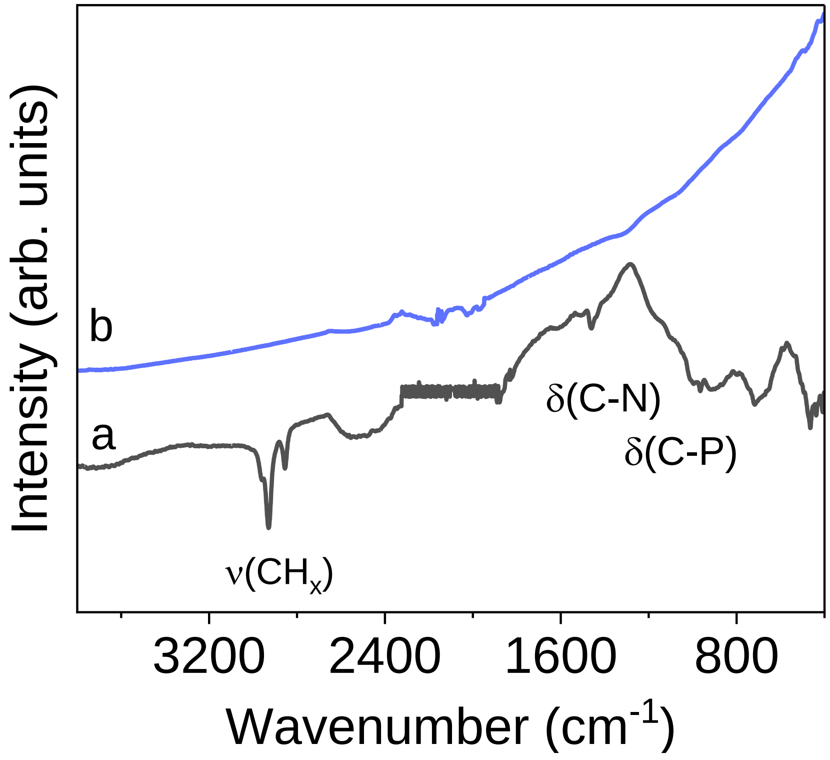

Figure S10. FTIR spectra of Ni_2P NCs (a) before and (b) after annealing at 450 °C for 2 h under 5% H_2:Ar atmosphere. Elimination of residual surface ligands is evident by the absence of the C-H stretches at 2938 and 2859 cm^{-1}, C-N stretch at 1468 cm^{-1}, and C-P stretch at 1068 cm^{-1} after annealing.

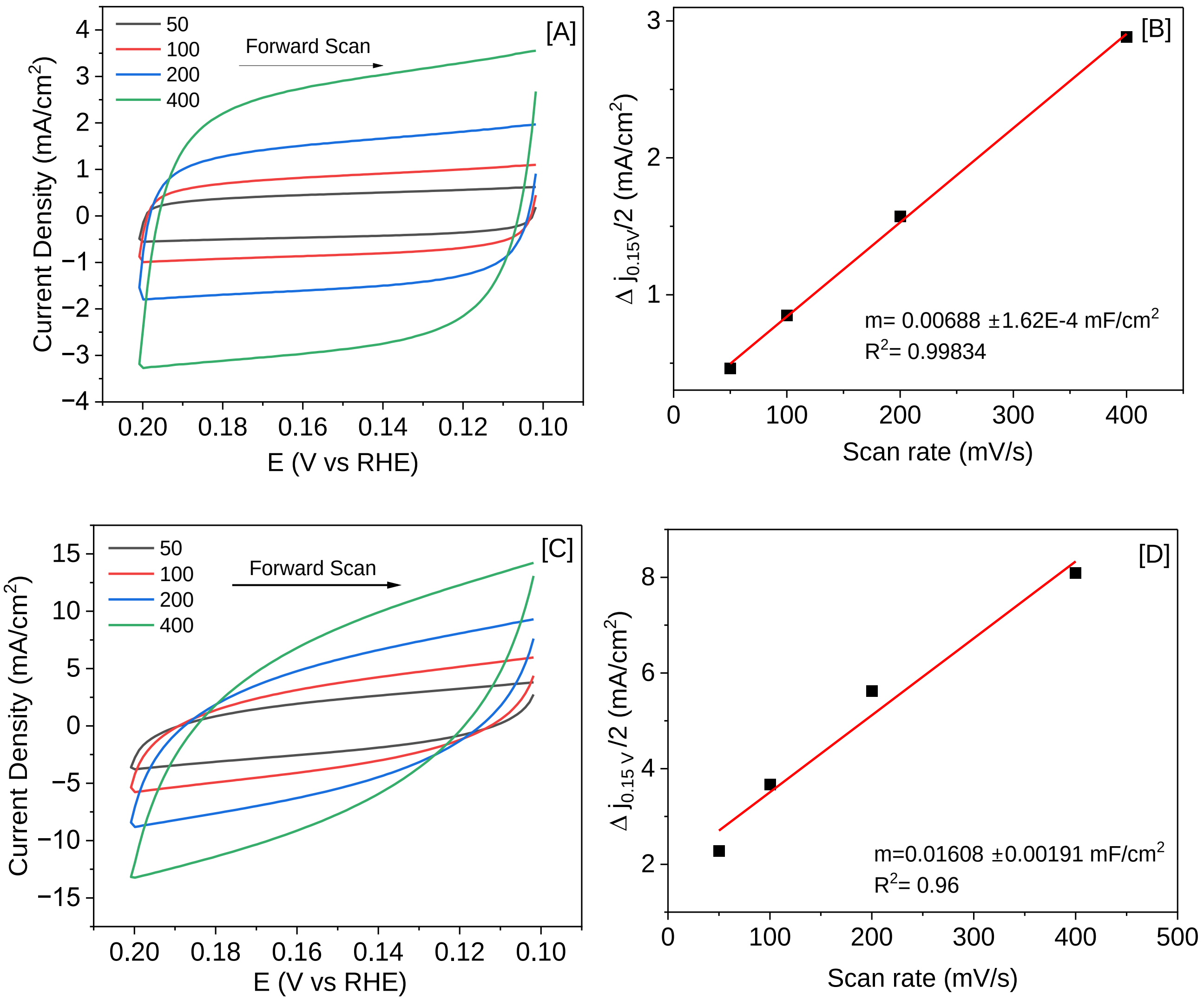

Figure S11. Calculation of the ECSA of $Ni_{2-x}Zn_xP$ NCs by estimating double-layer capacitance (C_{DL}) from cyclic voltammograms. [A] Cyclic voltammograms of Ni_2P and [B] corresponding C_{DL} plot along with [C] cyclic voltammograms of $Ni_{1.85}Zn_{0.15}P$ NCs and [D] subsequent C_{DL} plot. CVs were recorded at the scan rates of 50, 100, 200 and 400 mV/s in N_2-saturated 1 M KOH. Cathodic and anodic currents were measured at -0.15 V vs Hg/HgO and derived from their corresponding cyclic voltammograms as a function of scan rate. The difference in anodic and cathodic current densities were plotted vs the scan rate and the corresponding slope is the C_{DL}. The ECSA values were obtained by dividing the C_{DL} with the specific capacitance (Cs).

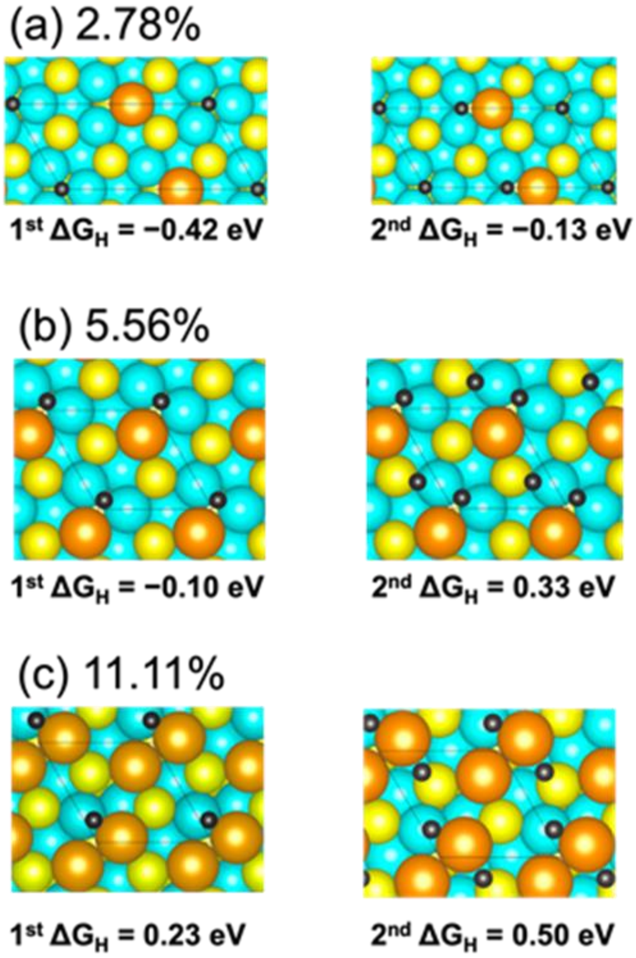

Figure S12. The lowest first and second ΔG_H on Zn-doped Ni_2P (001) with the Ni_3P_2 termination at Zn concentrations of 2.78%, 5.56%, and 11.11% calculated by PBE+D3(BJ).

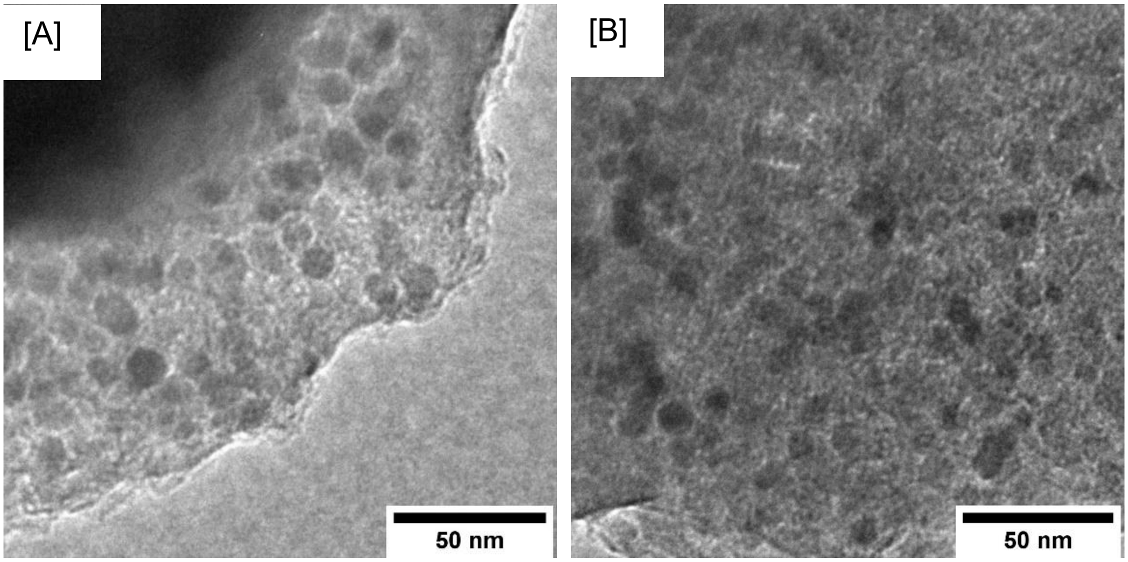

Figure S13. [A-B] Representative LR-TEM images of $Ni_{1.90}Zn_{0.10}P$ NCs showing no change in morphology after HER catalysis in 1 M KOH.

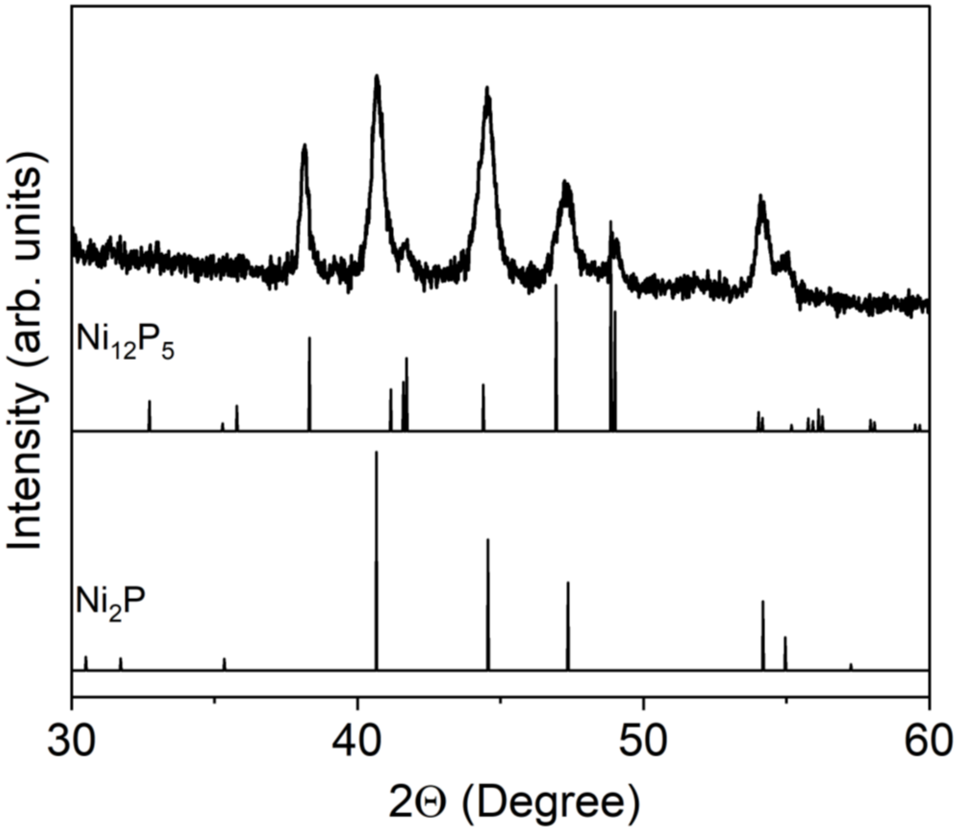

Figure S14. A representative PXRD pattern of $Ni_{1.90}Zn_{0.10}P$ NCs after catalysis. $Ni_{12}P_5$ (JCPDS No. 04-003-6050) and Ni_2P (JCPDS No. 01-074-1385) reference patterns are shown as black vertical lines.
